# Identification of twist-angle-dependent excitons in WS_2_/WSe_2_ heterobilayers

**DOI:** 10.1093/nsr/nwab135

**Published:** 2021-07-30

**Authors:** Ke Wu, Hongxia Zhong, Quanbing Guo, Jibo Tang, Jing Zhang, Lihua Qian, Zhifeng Shi, Chendong Zhang, Shengjun Yuan, Shunping Zhang, Hongxing Xu

**Affiliations:** School of Physics and Technology and Key Laboratory of Artificial Micro- and Nano-Structures of Ministry of Education, Wuhan University, Wuhan 430072, China; School of Physics, Huazhong University of Science and Technology, Wuhan 430074, China; School of Physics and Technology and Key Laboratory of Artificial Micro- and Nano-Structures of Ministry of Education, Wuhan University, Wuhan 430072, China; School of Physics and Technology and Key Laboratory of Artificial Micro- and Nano-Structures of Ministry of Education, Wuhan University, Wuhan 430072, China; The Institute for Advanced Studies, Wuhan University, Wuhan 430072, China; School of Physics and Technology and Key Laboratory of Artificial Micro- and Nano-Structures of Ministry of Education, Wuhan University, Wuhan 430072, China; School of Physics, Huazhong University of Science and Technology, Wuhan 430074, China; Key Laboratory of Materials Physics of Ministry of Education, School of Physics and Microelectronics, Zhengzhou University, Zhengzhou 450052, China; School of Physics and Technology and Key Laboratory of Artificial Micro- and Nano-Structures of Ministry of Education, Wuhan University, Wuhan 430072, China; School of Physics and Technology and Key Laboratory of Artificial Micro- and Nano-Structures of Ministry of Education, Wuhan University, Wuhan 430072, China; School of Physics and Technology and Key Laboratory of Artificial Micro- and Nano-Structures of Ministry of Education, Wuhan University, Wuhan 430072, China; School of Physics and Technology and Key Laboratory of Artificial Micro- and Nano-Structures of Ministry of Education, Wuhan University, Wuhan 430072, China; The Institute for Advanced Studies, Wuhan University, Wuhan 430072, China

**Keywords:** heterobilayers, twist angle, exciton, indirect transition, transition dipole moment

## Abstract

Stacking atomically thin films enables artificial construction of van der Waals heterostructures with exotic functionalities such as superconductivity, the quantum Hall effect, and engineered light-matter interactions. In particular, heterobilayers composed of monolayer transition metal dichalcogenides have attracted significant interest due to their controllable interlayer coupling and trapped valley excitons in moiré superlattices. However, the identification of twist-angle-modulated optical transitions in heterobilayers is sometimes controversial since both momentum-direct (K–K) and -indirect excitons reside on the low energy side of the bright exciton in the monolayer constituents. Here, we attribute the optical transition at ∼1.35 eV in the WS_2_/WSe_2_ heterobilayer to an indirect Γ–K transition based on a systematic analysis and comparison of experimental photoluminescence spectra with theoretical calculations. The exciton wavefunction obtained by the state-of-the-art GW-Bethe-Salpeter equation approach indicates that both the electron and hole of the excitons are contributed by the WS_2_ layer. Polarization-resolved *k*-space imaging further confirms that the transition dipole moment of this optical transition is dominantly in-plane and is independent of the twist angle. The calculated absorption spectrum predicts that the so-called interlayer exciton peak coming from the K–K transition is located at 1.06 eV, but with a much weaker amplitude. Our work provides new insight into the steady-state and dynamic properties of twist-angle-dependent excitons in van der Waals heterostructures.

## Teaser

The twist-angle-modulated excitons in vertically stacked WS_2_/WSe_2_ heterobilayers are proven to be an indirect G-K intralayer transition, with a nearly in-plane transition dipole moment.

## INTRODUCTION

Constructing heterostructures via van der Waals interactions mitigates the general requirement of lattice matching in epitaxially grown samples, enabling a large variety of metamaterials by stacking different thin layers. Additional degrees of freedom, such as the combinations of constituents or their twist angles, can be used to engineer the mechanical, electrical, magnetic, and optical properties of heterostructures [1–5]. In particular, vertically stacked transition metal dichalcogenide (TMD) heterobilayers have attracted significant interest because their type-II band alignment favors the creation of interlayer excitons [1,6–9] with ultralong lifetime [4] and valley depolarization time [10]. The twist angle and the mismatch in the lattice constants of the monolayers can create a periodic moiré potential as deep as 116 meV [11]. Interlayer excitons trapped in the moiré potential exhibit alternating circularly polarized photoluminescence (PL) originating from spatially varying optical selection rules within the moiré supercell [11,12]. These appealing properties of interlayer excitons make them an excellent platform for exploring Bose–Einstein condensation or a new carrier of quantum information in functional exciton devices.

However, identifying the origin of the new optical transitions in TMD heterobilayers is a tough task, and controversial conclusions have been continuously reported in the literature. For example, the peak at ∼1.35 eV in MoSe_2_/WSe_2_ heterobilayers was attributed to interlayer excitons, originating from the momentum-direct (K–K) transition, by many research groups [1,3,10–12], but was also called an indirect [13] or mixed transition [7], even though the experimental spectra look similar. The opposite circular polarization of the PL at different wavelengths, a key piece of evidence of the origin, was attributed to tilted electron spin [13] caused by hybridization of the electron between the layers or varied optical selection rules modulated by the moiré superlattice [3]. For other combinations, such as MoS_2_/WSe_2_ and MoSe_2_/WS_2_, the new optical transitions on the low energy side of the bright excitons in the monolayer constituents were proven to possess a strong intralayer character [8] or intra- and interlayer hybridized character [14]. The K–K transition was found in the infrared region at ∼1.0 eV [15] (note that the K–K transitions in heterobilayers are generally optically dark between the centers of the two valleys [16]). A detailed summary of the identifications of new optical transitions in TMD heterobilayers is presented in Supplementary Data 1.1. Recently, intense research has been devoted to the WS_2_/WSe_2_ combination, including the discovery of a pure spin-valley diffusion current [17], moiré-trapped excitons [18,19], and an ultrafast exciton phase transition [20]. The new optical transition at ∼1.45 eV observed in these works was called the interlayer exciton transition, which, without specific notation, refers to the K–K valley transition. In this work, we show that this optical transition, possessing a nearly in-plane (IP) transition dipole moment, originates from a momentum-indirect Γ–K transition and is contributed by WS_2_ only. This identification is based on a systematic analysis and comparison of experimental PL spectra, twist-angle-dependent density functional theory (DFT) band structure calculations, more accurate DFT-GW calculations, and state-of-the-art optical calculations using the GW-Bethe-Salpeter equation (GW-BSE) approach. The nearly IP nature of the transition dipole moment, obtained from polarization-resolved *k*-space imaging of the PL emission, is found to be independent of the twist angle. Our calculations also predict that the interlayer exciton peak from the K–K transition resides in the infrared region at ∼1.06 eV, similar to that in the MoS_2_/WSe_2_ combination. The identification of the transitions in TMD heterobilayers helps clarify the origin of the excitons in the moiré superlattice, and the characterization of their transition dipole orientation is critical for their excitation or collection efficiency and their integration with optical microcavities or waveguides.

## RESULTS AND DISCUSSION

**Figure 1.**
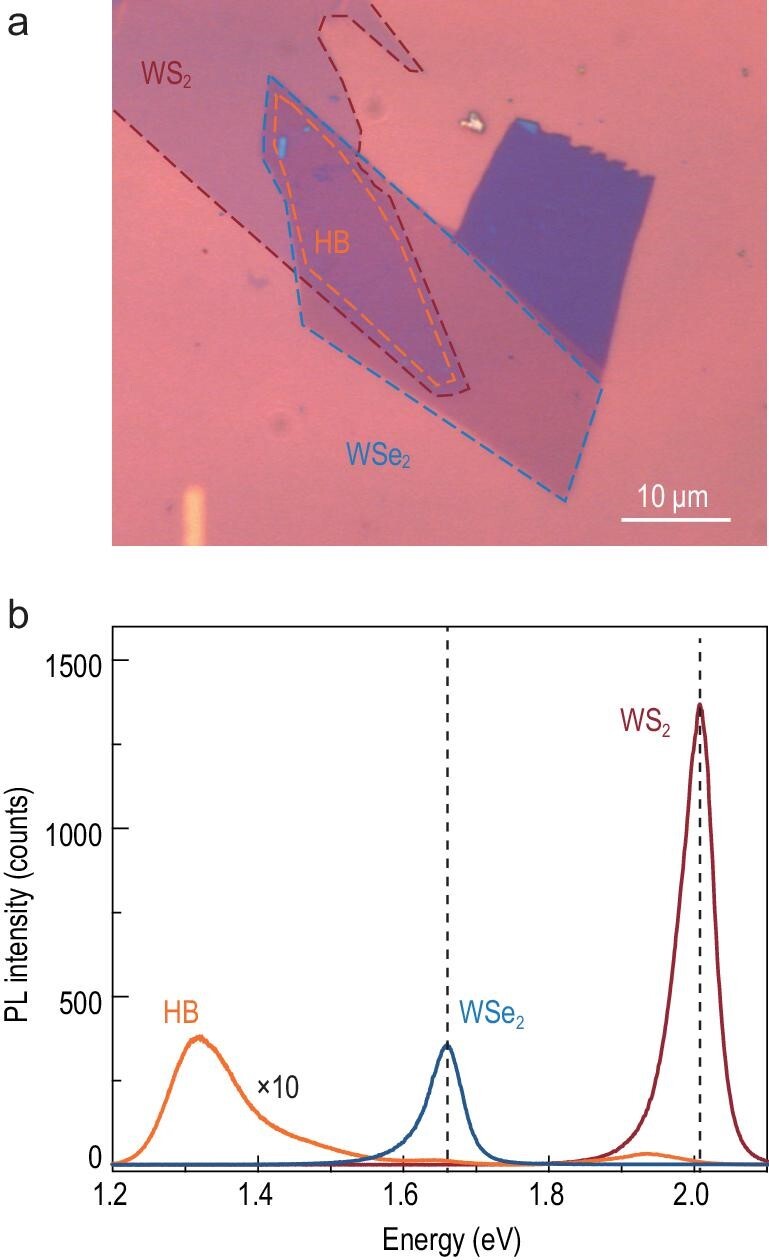
(a) Optical image of a 60° WS_2_/WSe_2_ heterobilayer (HB) on a Si substrate with 285 nm SiO_2_. The orange dotted line outlines the heterobilayer region. (b) PL spectra of monolayer WS_2_ (red), monolayer WSe_2_ (blue) and the heterobilayer (orange). The vertical dotted lines indicate the A exciton peak in the monolayers.

The heterobilayers were prepared by mechanical exfoliation, alignment, and stacking. An optical image of 60°WS_2_ (top)/WSe_2_ (bottom) heterobilayers on a 285 nm SiO_2_/Si substrate is shown in Fig. 1a. The twist angle was determined by polarization-resolved second-harmonic measurements [21]. The fabrication and optical characterization details can be found in Supplementary Data 1.2 and 1.3. Figure 1b shows the typical PL spectra taken from the 60°WS_2_/WSe_2_ heterobilayer and its monolayers. Monolayer WS_2_ and WSe_2_ show strong PL intensities at ∼2.0 eV and ∼1.65 eV, corresponding to their bright excitons. These two peaks are strongly quenched and redshifted in the heterobilayer, which is usually attributed to ultrafast interlayer charge transfer [22] and dielectric screening [1,23] from the adjacent layers, respectively. A new peak at ∼1.33 eV appears in the heterobilayer, which is absent in the monolayers, in agreement with previous reports [17,19,24].

**Figure 2.**
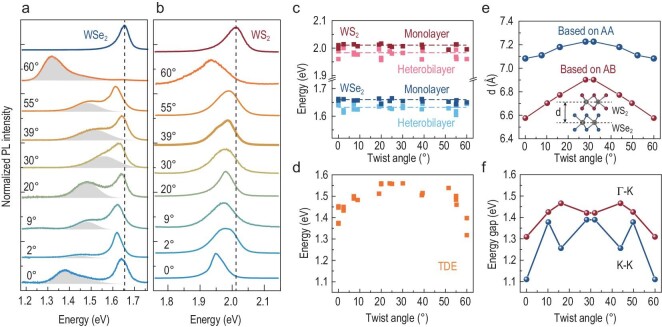
(a and b) PL spectra of monolayer WSe_2_ (blue), monolayer WS_2_ (red) and their heterobilayers with various twist angles (0° ≤ θ ≤ 60°). The PL spectra are normalized in the energy ranges of (a) 1.18–1.76 eV and (b) 1.76–2.15 eV. Vertical dotted lines indicate the bright exciton energies of the monolayers. (c) Energies of the bright exciton in monolayer WS_2_ (dark red), monolayer WSe_2_ (dark blue) and their corresponding heterobilayers (light red and light blue) as a function of twist angle. The mean value is plotted as a dashed line. (d) Energy of the TDE in the WS_2_/WSe_2_ heterobilayer as a function of twist angle. (e) Twist-angle dependence of the average layer distance of the WS_2_/WSe_2_ heterobilayer based on AA-stacking (blue) and AB-stacking (red) configurations, calculated by dispersion-corrected DFT. (f) Calculated K–K (blue) and Γ–K (red) transition energies for AB-stacked heterobilayers with different twist angles.

Now, we explore the energy variation of the excitons in the heterobilayer by controlling the twist angle. A total of 18 heterobilayers were fabricated with a twist angle ranging from 0° to 60°. The samples that showed a weak defect emission in the monolayer regions were selected. To facilitate the comparison, the PL spectra were normalized in the ranges of 1.18–1.76 eV (Fig. 2a) and 1.76–2.15 eV (Fig. 2b). The twist-angle-dependent PL peaks are highlighted by gray shadows. Convoluted Lorentzian and Gaussian line shapes were used to fit the spectra, and the fitting accuracy of all spectra was greater than 0.998. Representative fitting examples of 30° and 0° heterobilayer samples are shown in Fig. S5. The bright exciton peaks of WS_2_ and WSe_2_ in the heterobilayer do not exhibit any dependence on the twist angle except for an overall 20–30 meV redshift compared to the monolayer peaks (Fig. 2c). Due to the staggered band alignment of the heterobilayers, the electrons (holes) tend to accumulate in the conduction (valence) band in WS_2_ (WSe_2_). Therefore, the original n-type WS_2_ and p-type WSe_2_ are further doped, which causes a higher trion ratio and a larger energy shift of the bright excitons in WS_2_ and WSe_2_. The peak of the new exciton in the heterobilayer shifts continuously from 1.35 eV to 1.57 eV as the twist angle varies from 0° to 30° (Fig. 2d). The energy of this twist-angle-dependent exciton (TDE) is highest at 30° and then decreases to the lowest energy at 0° or 60°. A similar twist-angle dependence of PL spectra has also been found in MoS_2_/WSe_2_ [8], MoSe_2_/WS_2_ [14] and twisted bilayer MoS_2_ [25,26]. This phenomenon has been attributed to the symmetry changes of the layer spacing and transition energy with the twist angle [8], which are also explored in Fig. 2e and f. The energy of the TDE in the nearly aligned sample is lower than that in previous reports [18,19], which show a value of ∼1.45 eV. We think it is the naked sample structure (without hBN protecting), the room measuring temperature and the exact stacking angle of the nearly aligned heterobilayers that lead to the much lower energy of the TDE in WS_2_/WSe_2_. As shown in Fig. 2a, once the twist angle shifts from 0° to 2°, the energy of the TDE shifts from ∼1.35 eV to ∼1.45 eV. Then, we focus on the K–K and Γ–K optical transitions that are affected by the van der Waals interlayer interaction. With an increase in the twist angle from 0° to 60°, both the layer distance (Fig. 2e) and Γ–K transition energy (Fig. 2f) increase, reach their maximum near 30° and then decrease to lower values. By comparison, the variation in the K–K transition energy with twist angle is not consistent with the experimentally observed TDE results. Particularly, at 0° (60°) and 16° (44°), there is a 200–300 meV difference between the experimental and theoretical results. Although this comparison indicates that the Γ–K transition is closer to the experimental observations, one cannot exclude the possibility of a K–K transition based solely on the twist-angle dependence. More theoretical analysis will be present below. We also consider the strain effect on the energy gap (see Fig. S13). The results show that a smaller strain leads to a larger energy gap. Moreover, in experiments, the energy of the TDE varies by ∼220 meV when the twist angle changes from 0° to 30°, which is much larger than that of the TDE in MoS_2_/WSe_2_ [8]. The large fluctuation of TDE energy is caused by the larger variation in the interlayer distance (0.30 Å) in WS_2_/WSe_2_ heterobilayers compared to that (0.07 Å) in MoS_2_/WSe_2_ heterobilayers. These different variation degrees of the interlayer distance are related to the different van der Waals force interactions caused by the metal cations in WS_2_ and MoS_2_.

**Figure 3.**
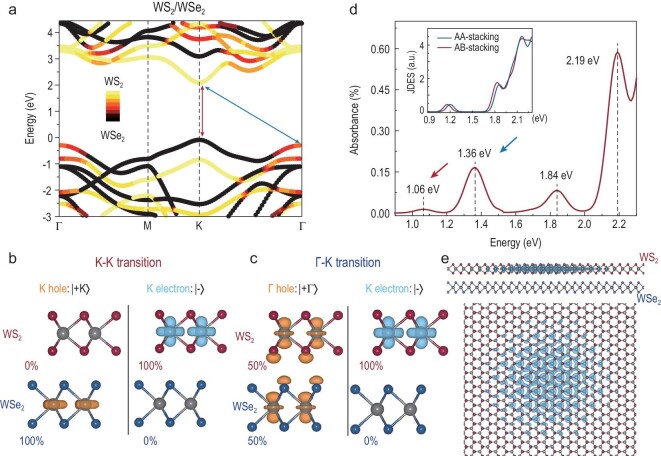
(a) Projected band structure of the AB-stacked WS_2_/WSe_2_ heterobilayer obtained by the GW approach. The color bar indicates the projection of the wavefunction on each layer. Two low-energy exciton peaks from the valence band to the conduction band are labeled by red and blue arrows. Distribution of the hole | +〉 and electron | −〉 states associated with (b) K–K excitation and (c) Γ–K excitation. The electron state | −K〉 is distributed only in the WS_2_ layer, while the hybrid hole | +Γ〉 state is distributed equally in both layers. (d) Average optical absorption spectra of AA- and AB-stacked WS_2_/WSe_2_ heterobilayers, calculated via DFT-GW-BSE with Gaussian smearing of 50 meV. The insert plots are the calculated joint density of excited states for primitive AA-stacked (blue line) and AB-stacked (red line) WS_2_/WSe_2_; only direct optical transitions are included in this calculation. (e) Real-space distribution of the charge density in the TDE. The hole is fixed in the WS_2_ layer. Top: side view. Bottom: top view.

In the following, based on first-principles calculations, we further explore the origin of the TDE in the WS_2_/WSe_2_ heterobilayer by analyzing the excitonic weighting factor. Figure 3a shows the projected type-II band structure of an AB-stacked WS_2_/WSe_2_ heterobilayer, where the conduction band minimum (valence band maximum) at the K point belongs to the WS_2_ (WSe_2_) layer (for AA stacking, see Supplementary Data 2.3). This is consistent with the partial charge densities of the electron and hole states in K–K and Γ–K transitions, as indicated in Fig. 3b and c. Three states are involved in these transitions: the K-electron state | −K〉, the K-hole state | +K〉 and the Γ-hole state | +Γ〉. For the Γ–K transition, | +Γ〉 is strongly affected by the interlayer hybridization and is distributed equally in both layers, i.e. half in the WS_2_ layer and half in the WSe_2_ layer. To intuitively analyze the origin of the observed TDE, we present the average optical absorbance of AA- and AB-stacked WS_2_/WSe_2_ heterostructures including the excitonic effects [22] in Fig. 3d (see details in Fig. S17). Using excitonic weighting factor analysis, the intralayer ‘strongly absorbing’ excitons at 1.84 eV and 2.19 eV belong to WSe_2_ and WS_2_, respectively (Table S3). The low-lying peak at 1.36 eV originates from the Γ–K transition, corresponding to the experimentally observed TDE. The indirect nature of the 1.36 eV peak can be verified by the disappearance of the zero joint density of excited states at ∼1.36 eV (Fig. 3d and Fig. S18); the peak at 1.36 eV disappears when only the direct optical transition is included in the calculation. In recent reports, this kind of TDE formed by a hybrid hole or electron state was named a hybrid exciton [14,15,27,28]. Figure 3e shows that the electronic part of the exciton wavefunction will localize in the WS_2_ layer when the hole is fixed in the WS_2_ layer. This figure indicates that the TDE comes from the intralayer transition in WS_2_. In addition, the near-infrared exciton peak at 1.06 eV is identified as the real interlayer exciton peak, coming from the K–K transitions between the two layers [15]. In the experiment, although we tried to find this interlayer exciton PL as efficient as possible, even by tilting the sample 45°, we did not observe any PL signal in the near-infrared region from 0.83 eV to 1.13 eV (not shown here). This is attributed to its relatively small amplitude caused by the spatial separation of electron and hole states, as shown in Fig. 3b. Such weak near-infrared exciton emission at room temperature has been observed in MoS_2_/WSe_2_ heterobilayers [15].

**Figure 4.**
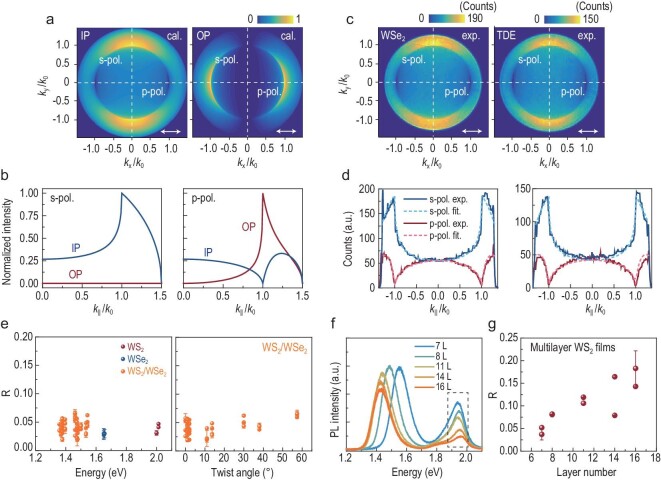
(a) Calculated and normalized *k*-space emission patterns of the pure IP (left) and OP (right) dipoles. The dipole is located in an infinitesimally thin film, emitting at 1.35 eV and sitting on a quartz substrate (*n* = 1.5). The white arrows denote the x-polarization direction and dashed lines denote s- and p-polarized cross sections. (b) Cross sections for s- and p-polarization of IP (left) and OP (right) dipoles. (c) X-polarized *k*-space emission pattern of the bright (A) exciton (left) in monolayer WSe_2_ at 1.65 eV and TDE (right) at ∼1.35 eV. The corresponding experimental and fitting cross sections for s- and p-polarization are shown in (d): left, WSe_2_; right, TDE. (e) Left: ratio *R* of the bright exciton in monolayers and TDE in heterobilayers as a function of the exciton energy. Right: ratio *R* of the TDE as a function of twist angle. (f) PL spectra of the multilayer WS_2_ films. The *k*-space pattern of the A exciton of multilayer WS_2_ at ∼1.95 eV marked by a dotted box was measured. (g) Ratio *R* of the A exciton in (f) as a function of the layer number of multilayer WS_2_.

The exciton wavefunction of the TDE in WS_2_/WSe_2_ is similar to that of the bright excitons in monolayer TMDs, which are IP excitons [29–31]. We used the back focal plane imaging (Fourier imaging) technique to quantify the orientation of the transition dipole moment of the TDE in the WS_2_/WSe_2_ heterobilayer [32–34]. This optical dipole characterizes the magnitude of the optical transition between the ground state | 0〉 and the exciton state | X〉 [2]. In Fig. 4a, we show the simulated *k*-space emission patterns of a pure IP (left) and out-of-plane (OP) (right) dipole (emitting at 1.35 eV) positioned on a quartz substrate (refractive index of 1.5). In the Fourier image, every point corresponds to an IP momentum *k*_||,_ which equals *nk*_0_ sin*θ*, where *k*_0_ is the wavenumber in air, *n* is the refractive index of oil (1.5) and *θ* is the light emission angle in oil. The light can be decomposed into p-polarized and s-polarized light. In the calculation, the electric field is projected onto the x-axis, corresponding to the usage of a polarizer in front of the back focal plane in the experiment. This treatment ensures that the light intensity along *k*_y_ = 0 (*k*_x_ = 0) reflects the p-component (s-component) of the emitted light. More details about the Fourier model can be found in Supplementary Data 1.4. The pure IP dipole can radiate in both the s- and p-polarization directions, whereas the pure OP dipole shows a vanishing radiation intensity along the s-polarization direction. At the critical angle (*k*_||_ = *k*_0_), the intensities of p-polarized light of the IP and OP dipoles reach their minimum and maximum, respectively (Fig. 4b). The entire angular distribution of the radiation intensity enables us to decompose the intensity along *k*_y_ = 0 into the contributions of an IP and/or OP dipole.

In the experiment, we excite and collect the signals from the back of the quartz substrate. The PL intensity distribution at the back focal plane of the objective (N.A. = 1.4) is projected onto a charge-coupled device (CCD) camera. The s- and p-polarized light is analyzed by using x- and y-oriented polarizers, respectively. Figure 4c (left) shows the polarization-resolved *k*-space emission pattern of the bright exciton from monolayer WSe_2_ centered at 1.65 eV with a filter bandwidth of 63 meV (13 nm). One can easily note that the pattern of the bright exciton resembles the distribution for a pure IP dipole situation illustrated in Fig. 4a (left) [32–34]. The cross section profiles along *k*_y_ = 0 and *k*_x_ = 0 reveal that the OP component is negligible for the A exciton of monolayer WSe_2_, which is also confirmed by the nearly vanishing PL intensity at *k*_||_ = *k*_0_ for p-polarization (Fig. 4d (left)). As we discuss in Supplementary Data 1.4, the results are the same for the bright exciton in monolayer WS_2_ [32,33]. Similarly, the *k*-space emission pattern of the TDE at ∼1.35 eV also presents the IP dipole character, which is also confirmed by the fitting results (Fig. 4c (right), Fig. 4d (right)). In our imaging experiments, we note that the PL intensity at the critical angle for p-polarization is close to the noise level of the CCD.

Moreover, the polarization-resolved *k*-space emission patterns of TDEs in 0° to 60° samples were investigated. The fittings to the TDE results show a dipole orientation in the range of 89–90°, restricted by the background noise of the CCD. To allow a better quantification of the dipole orientation, we define the ratio *R* = *I*_p_ (*k*_||_ = *k*_0_)/*I*_s_ (*k*_||_ = *k*_0_), where *I*_p_ and *I*_s_ are the emission intensities for p- and s-polarization at *k*_||_ = *k*_0_ in the x-polarized *k*-space pattern. *R* increases from zero to infinity as the dipole changes from IP (90°) to OP (0°). In principle, the ratio *R* can be used to precisely determine the dipole orientation, as shown in Supplementary Data 1.4. However, the experimental value of *R* for the pure IP exciton (bright exciton) of monolayer TMDs is ∼0.03 due to the noise of the CCD, as shown in Fig. 4e. Therefore, we compare the ratio of the TDEs with that of the bright excitons in the monolayers to determine the dipole orientation. For the TDE in heterobilayers, the ratio *R* shows no obvious dependence on the emission energy (Fig. 4e (left)) or twist angle (Fig. 4e (right)), also fluctuating around 0.03, which is comparable to the ratio of the bright excitons in monolayers. Interestingly, a small OP transition contribution to the A exciton in multilayer WS_2_ (which is an indirect exciton) can be detected using back focal plane imaging. Figure 4f shows the PL spectra of multilayer WS_2_. The *k*-space pattern of the A exciton at ∼1.95 eV, marked by the dotted box, was measured. The *R* of this exciton increases from 0.03 to ∼0.2 as the number of layers increases from 7 to 16 (Fig. 4g). The fitting results show that the CCD can distinguish excitons with an 84° (*R*∼0.12) orientation (Supplementary Data 1.4). This phenomenon can be supported by previous theoretical calculations [35], which predicted that for the A excitons in bulk TMDs, when the hole is fixed in one layer, the probability of finding an electron in the adjacent layer is ∼8% [35]. Based on these results, we deduce that IP excitons account for the majority of the TDEs in WS_2_/WSe_2_ heterobilayers and that the dipole orientation is in the range of 85∼90°.

## CONCLUSION

In conclusion, we performed a combined experimental and state-of-the-art theoretical study of the TDE in WS_2_/WSe_2_ heterobilayers. The peak energy of the exciton varies symmetrically with the twist angle, centered at 30°. By comparison with the calculated results, we attribute the optical transition at 1.35 eV to the indirect Γ–K transition, where the Γ hole state is a hybrid state. Theoretical calculations based on the DFT-GW-BSE approach further confirm the intralayer character of this TDE, with the electron and hole both contributed by the WS_2_ layer. The nearly IP oriented transition dipole character of the TDE (85–90°), revealed by the *k*-space emission pattern, is independent of the twist angle. Our calculations also predict that the exciton peak coming from the K–K transition is located at ∼1.06 eV. Identifying the origin of the TDE in heterobilayers is essential to the understanding of these quasiparticles. Characterizing their transition dipole moment is crucial for further design of high-efficiency optoelectronic and nanophotonic devices based on van der Waals heterostructures.

## METHODS

### Sample fabrication

WS_2_/WSe_2_ heterobilayers were prepared using a standard PDMS/PVA stamping method (PDMS: polydimethylsiloxane; PVA: polyvinyl alcohol). First, monolayer WS_2_ and monolayer WSe_2_ were mechanically exfoliated from bulk crystals (HQ graphene) and deposited onto a Si substrate with 285 nm SiO_2_. Second, stacked polymer films of PDMS/PVA (PDMS: top; PVA: bottom) were coated on monolayer WS_2_ and heated to 70$^\circ {\rm{C}}$. After cooling, the monolayer WS_2_ was separated from the substrate together with the PDMS/PVA film. Third, the PDMS/PVA/monolayer WS_2_ film was coated on the target monolayer WSe_2_. Repeating the heating and cooling steps, the PVA/WS_2_/WSe_2_ structure remained on the substrate after tearing off the PDMS. Finally, WS_2_/WSe_2_ could be obtained by dissolving the PVA film in water. For WS_2_/WSe_2_ on a quartz substrate, in the third step, PVA/WS_2_/WSe_2_ was torn off from the SiO_2_/Si substrate and stamped onto the quartz substrate before dissolving the PVA film. The above transfer process was carried out under a microscope and micromanipulation platform. All the heterobilayers were annealed at 300$^\circ {\rm{C}}$ in argon at atmospheric pressure for 3 hours.

### PL characterization

All the experiments in the manuscript were conducted at room temperature. The PL spectra of the samples were excited by a continuous-wave 532 nm laser and collected by a 100× objective (air: MPLFLN-BD, Olympus, NA = 0.9). The signal was dispersed by a 300 lines/mm blazed grating and sent to a microspectrometer (Renishaw inVia). More detail on the Fourier imaging and fitting model can be found in the Supplementary Data.

## Supplementary Material

nwab135_Supplemental_File
