# Supplementary material for: Identification of twist-angle-dependent excitons in WS2/WSe2 heterobilayers
Source: Natl Sci Rev. 2021 Jul 30;9(6):nwab135. doi: 10.1093/nsr/nwab135 (PMC9252742; doi:10.1093/nsr/nwab135)
Supplement: nwab135_Supplemental_File [file nwab135_supplemental_file.docx]

**Supporting Information For**

**Identification of twist-angle-dependent excitons in WS_2_/WSe_2_ heterobilayers**

Ke Wu,^1, 2, †^ Hongxia Zhong,^1, †^ Quanbing Guo,^1^ Jibo Tang,^3^ Jing Zhang,^1^ Lihua Qian,^2^ Zhifeng Shi^4^, Chendong Zhang,^1^ Shengjun Yuan,^1, *^ Shunping Zhang,^1, *^ and Hongxing Xu^1, 3, *^

^1^School of Physics and Technology and Key Laboratory of Artificial Micro- and Nano-structures of Ministry of Education, Wuhan University, Wuhan 430072, China.

^2^School of Physics, Huazhong University of Science and Technology, Wuhan 430074, China.

^3^The Institute for Advanced Studies, Wuhan University, Wuhan 430072, China.

^4^Key Laboratory of Materials Physics of Ministry of Education, School of Physics and Microelectronics, Zhengzhou University, Daxue Road 75, Zhengzhou 450052, China

^*^e-mail: [spzhang@whu.edu.cn](mailto:spzhang@whu.edu.cn); [s.yuan@whu.edu.cn](mailto:s.yuan@whu.edu.cn); [hxxu@whu.edu.cn](mailto:hxxu@whu.edu.cn).

**Contents**

[I Supporting experimental details 3](#_Toc64123067)

[1.1 Summary of the electronic properties of twist-angle dependent excitons (TDEs) in recent reports 3](#_Toc64123068)

[1.2 Optical photoluminescence (PL), Fourier imaging, atomic force microscopy (AFM) and second-harmonic generation (SHG) measurements. 3](#_Toc64123069)

[1.3 Fourier model and Fourier images of excitons in monolayers and HBs. 11](#_Toc64123070)

[II Supporting theoretical details 18](#_Toc64123071)

[2.1 Density functional theory (DFT) and GW calculation methods. 19](#_Toc64123072)

[2.2 Twisted WS_2_/WSe_2_ HBs 20](#_Toc64123073)

[2.3 Electronic structures of AA- and AB-stacked HBs 27](#_Toc64123074)

[2.4 Optical transitions 28](#_Toc64123075)

[2.4.1 Moiré pattern 28](#_Toc64123076)

[2.4.2 Optical absorbance 29](#_Toc64123077)

[2.4.3 Joint density of excited states of WS_2_/WSe_2_ 29](#_Toc64123078)

[2.4.4 Excitonic weight analysis 31](#_Toc64123079)

## **I Supporting experimental details**

### 1.1 Summary of the electronic properties of twist-angle-dependent excitons (TDEs) in recent reports

**Table S1** Momentum space and real space nature of TDEs in recent works.

|  | Momentum space transition | Type of low energy exciton | Energy (eV) | References |
| --- | --- | --- | --- | --- |
| MoSe_2_/WSe_2_ | K-K | Interlayer | ~1.3-1.4 | Xu and Yao’s group [1-3]  Xiaoqin Li et al. [4]  Hyeon Suk Shin et al. [5] |
|  | K-K and Σ-K | Interlayer | ~1.3-1.4 | Ursula Wurstbauer et al. [6] |
|  | Q-K | Not purely interlayer | ~1.3-1.4 | Berend T. Jonker et al. [7] |
| MoS_2_/WSe_2_ | Γ-K | Strong monolayer character in MoS_2_ | ~1.6 | Tobias Korn et al. [8] |
|  | K-K and Γ-K | K-K: interlayer  Γ-K: mixed inter- and intralayer | 1.0~1.05  ~1.6 | Tony F. Heinz et al. [9] |
| MoSe_2_/WS_2_ | Γ-X_A_ | Mixed inter- and intralayer | 1.5-1.6 | Alexander I. Tartakovskii et al. [10] |
| WSe_2_/WS_2_ | K-K | Interlayer | 1.45-1.55 | Feng Wang et al. [11] |

### 1.2 Optical photoluminescence (PL), Fourier imaging, atomic force microscopy (AFM) and second-harmonic generation (SHG) measurements

Figure S1a shows the optical path for Fourier imaging and SHG measurements. A mirror can be flipped to send the signal to the Fourier imaging path (dotted line frame) or the SHG path. In the Fourier imaging path, a set of lenses is used to send the back focal plane image of the 100× objective (oil: Olympus, UPlanFLN, N.A. = 1.4) to the charge-coupled device (CCD: Q-imaging, Rolera EM-C2). The excitation light (continuous wavelength, 532 nm) is cleaned by a longpass filter of 590 nm, and the desired wavelength signal is obtained by a tunable bandpass filter (Semrock, bandwidth of approximately 13 nm). One exception is that when measuring the TDE that emits at 1.35 eV, the energy range of the filter is from 1.21 eV to 1.38 eV (TLP01-995-25×36). We measured the polarization response of the CCD, and its influence on the experimental results can be neglected. The optical path used in the PL Fourier imaging measurement is the part outside the dotted line frame. The spectrometer is an iHR320 (Horiba Jobin Yvon). Figure S1b shows the schematic of the Fourier imaging. In the experiment, the sample is placed in air, and the back of the substrate is immersed in oil. The signal is collected by a 100× objective (air: Olympus, MPLFLN-BD, N.A. = 0.9). The dipole orientation (α) is defined as the angle between the dipole axis and the z-axis. We can obtain the in-plane (IP) momentum (*k*_||_) at the back focal plane.

The second-harmonic optical path is the same as the PL spectra path in the Fourier imaging. In the SHG measurements, the sample is excited by a 796 nm pulsed laser with horizontal polarization (repetition rate of 79 MHz), and the second-harmonic signal is collected by a 100× objective (air: MPLFLN-BD, Olympus, N.A. = 0.9) with the same polarization as the excitation laser. The maximum SH intensity direction is the armchair direction of the monolayers [12]. In the text, the twist angle of the heterobilayers (HBs) is defined as the angle between monolayer WS_2_ and monolayer WSe_2_ in the armchair direction (Figure S2).


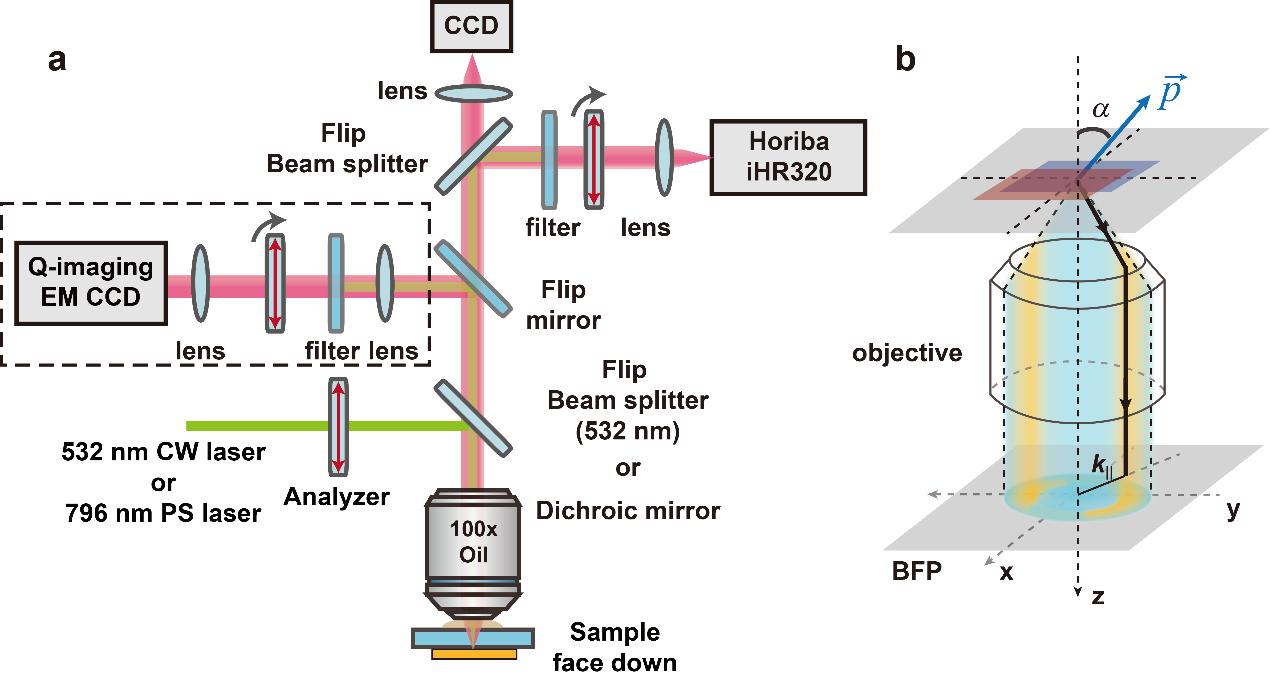
**Figure S1.** (a) Experimental setup for Fourier imaging (the dotted frame part) and SHG measurement. (b) Schematic of Fourier imaging. The signals are collected by an oil objective.


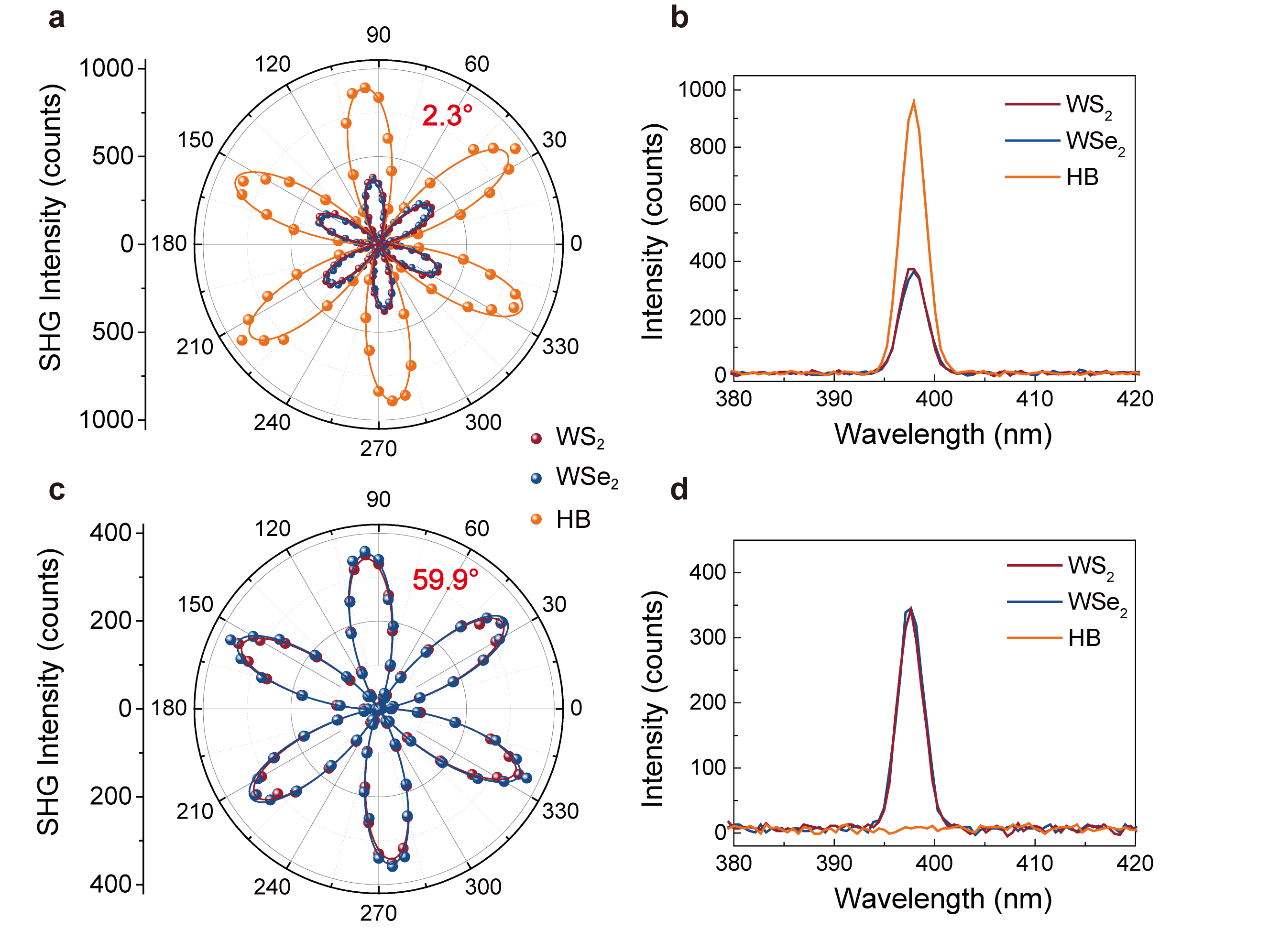


**Figure S2.** Polarization-resolved second-harmonic intensity from 2.3° (a) and 59.9° (c) stacked WS_2_/WSe_2_ HBs. The detected polarization of second-harmonic radiation components is parallel to that of the incident pulse laser (796 nm). (b) and (d) Second-harmonic spectra of monolayer WS_2_, monolayer WSe_2_, and the HBs in (a) and (c) for the same experimental configuration. The accuracy of the twist angle measurements is approximately ±1°.


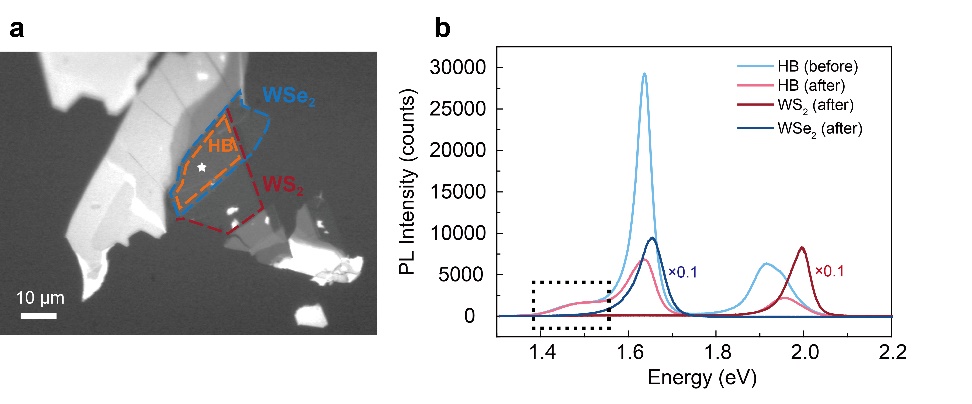
Before annealing, the PL peak of the TDE had already appeared (dotted rectangle). The peak intensity and shape of the bright excitons changed significantly compared to those obtained for the monolayers. After the annealing process, the intensity of the PL peak close to the those of the bright excitons in the monolayers was further quenched in the HB compared to the monolayers. The width of the PL peak close to that of the bright exciton in WS_2_ decreased, and the peak position blueshifted. These results indicate that there are two peaks in this region and that the lower-energy peak is quenched during annealing. Therefore, we believe that annealing can eliminate the influence of the transfer process on the quality of the sample (such as the doping effect or strain effect) and enhance the interlayer coupling. There is no obvious change in the TDE PL spectrum (including the spectrum shape and position) after annealing.

Figure S3. (a) Optical image of a WS_2_/WSe_2_ HB. (b) PL spectra before and after annealing, obtained at the star region marked in (a). The power of the excitation laser and the collection time were kept the same in all the acquisitions.

Figure S4a shows an optical image of a 1° WS_2_/WSe_2_ HB. In the red rectangular region of the optical image, the three blue particles are identified as large bubbles in the AFM image (three red circles in Figure S4b). In the PL measurement, we can avoid the large bubble area with the help of the microscope. The small bubbles shown in the AFM image are typically included in the PL measurements. Figure S4c characterizes the blue rectangular area in Figure S4a. The 0.76 nm and 0.75 nm heights of monolayer WSe_2_ and WS_2_ can be clearly distinguished in the enlarged AFM image. No impurities can be found in the characterization.


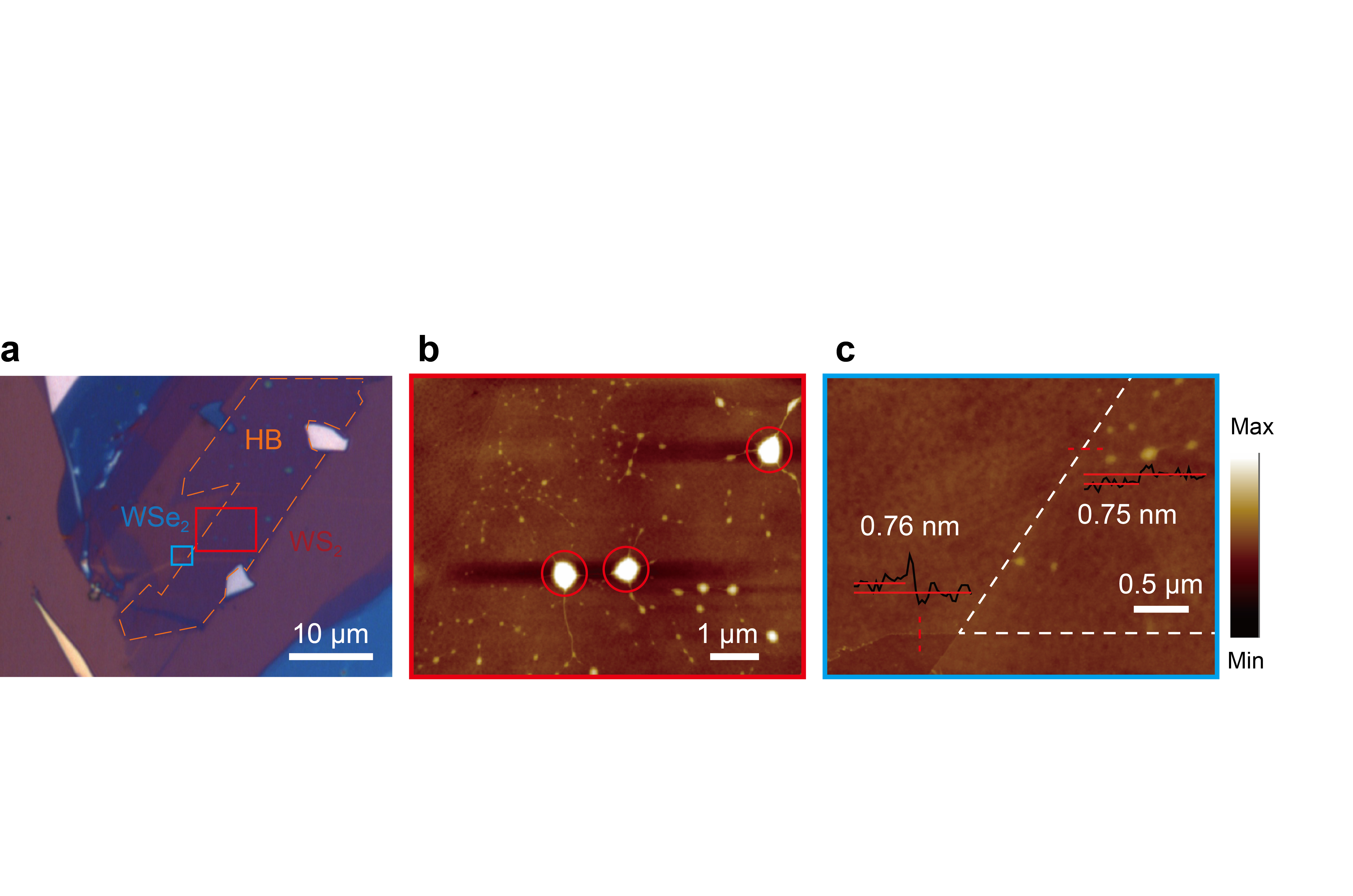


Figure S4. (a) Optical image of a 1° WS_2_/WSe_2_ sample. (b, c) AFM characterization associated with the red (b) and blue (c) rectangular areas in (a).


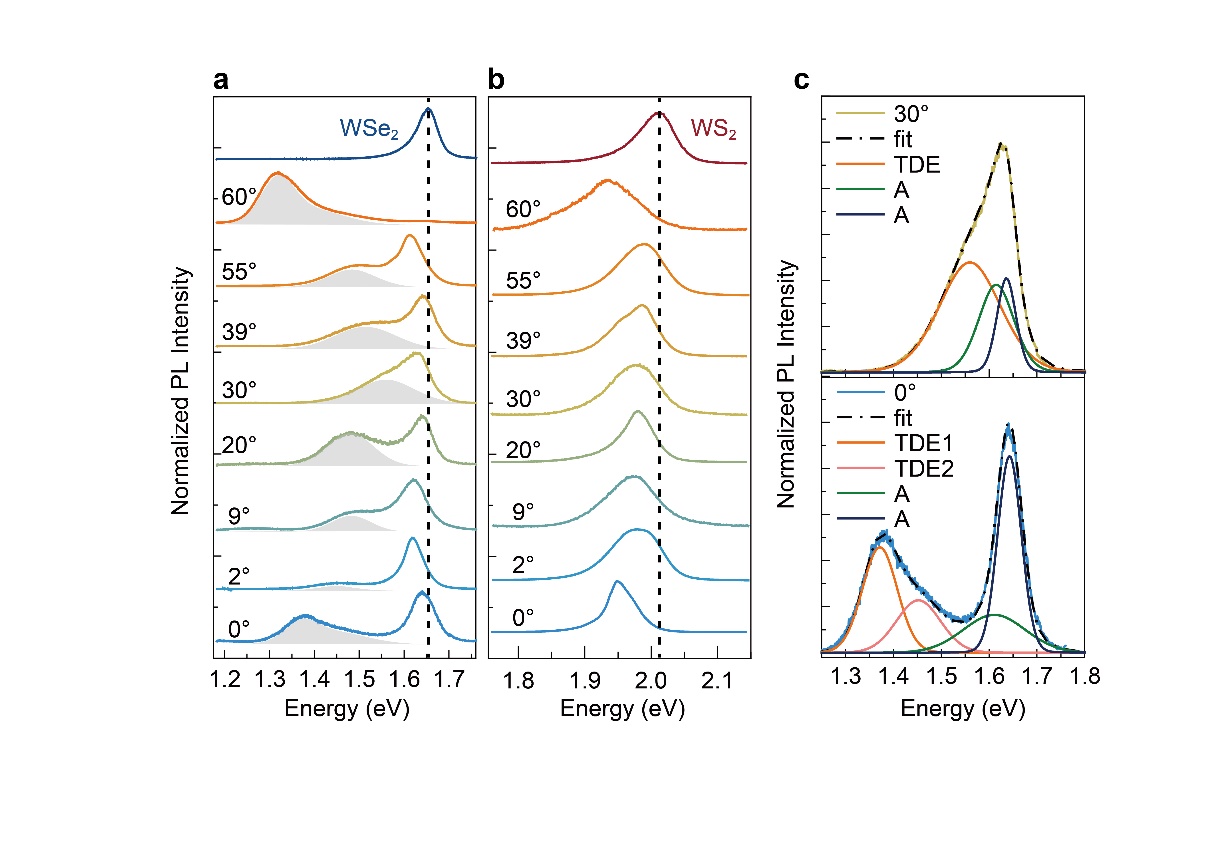
In the fitting process of the bright excitons in WSe_2_ and WS_2_, the energy space between the neutral exciton (A_0_) and trion was kept in the range of 20~60 meV. The TDEs can be well fitted by one peak except in the 0° or 60° sample. For the fitting example of the 0° sample, the TDE shows an asymmetric line, and two peaks are needed for fitting: one peak is at 1.37 eV, and the other peak is at 1.45 eV. In the statistical data, we include these two peaks in the statistics, as shown in Figure 2d. The TDE at approximately 1.40 to 1.45 eV can be found in HBs with 0<|θ-30°|<5°. We cannot distinguish this peak well in this work, and we deduce that it may come from a high TDE state in 0° or 60° samples according to previous PL excitation experiments on a WS_2_/WSe_2_ HB [11]. For the TDE in 0° or 60° samples, the energy range in the Fourier imaging is from 1.21 to 1.38 eV.

**Figure S5.** PL spectra fitting example of 0° (bottom) and 30° (top) HBs (original spectra are shown in Figure 2a).

### 1.3 Fourier model and Fourier images of excitons in monolayers and HBs

The analytical framework for infinitesimally thin films of Jon A. Schuller’s work [13] was adopted. The measured PL emission counts $N^{s,p}(\omega,k_{||})$ can be decomposed into contributions of the IP and out-of-plane (OP) dipole emissions [13]:

$$N^{s,p}\left( \omega,k_{\|} \right)=C_{exp}C_{0}\left( \omega\right)[\tilde{\rho}_{IP}^{s,p}\left( \omega,k_{\|} \right)\bar{n}_{IP}\left| \mu_{IP}\left( \omega\right) \right|^{2}+\tilde{\rho}_{OP}^{s,p}\left( \omega,k_{\|} \right)\bar{n}_{OP}\left| \mu_{OP}\left( \omega\right) \right|^{2}]$$

$C_{0}\left( \omega\right)=\frac{\omega^{3}}{c^{3}\hbar\pi\varepsilon_{0}}$ (2)

$C_{\exp}$is a constant that depends on the experimental parameters. $\text{C}_{0}(\omega)$is a function of $\omega$, which can also be set as a constant according to the exciton peak position. $\tilde{\rho}_{IP}^{s,p}\left( \omega,k_{\|} \right)$ ($\tilde{\rho}_{OP}^{s,p}\left( \omega,k_{\|} \right)$) is the local density of optical states (LDOS) for the IP (OP) dipole, $\bar{n}_{IP}$ ($\bar{n}_{OP}$) is the time-averaged population of the IP (OP) excitons, and $\mu_{IP}(\omega)$ ($\mu_{OP}(\omega)$) is the IP (OP) dipole moment of an emitter. $k_{x}=0$ and $k_{\text{||}}=k_{y}$when the PL emission is considered to be within the y-z plane. The coordinates and variables are defined as follows: the *z*-axis is normal to the substrate surface, the *x-* and *y*-axes are within the substrate IP, $\lambda$is the free space wavelength, $k_{0}=2\pi/\lambda$is the total free space momentum, $k_{zi}$is the momentum component along the z-axis in layer i, $n_{i}$ is the refractive index of layer i, and $t_{ij}^{p}$, $r_{ij}^{p}$and $r_{ij}^{s}$ are the p-polarized transmission coefficient, p-polarized reflection coefficient, and s-polarized reflection coefficient from layer i into layer j. For a given value of $k_{y}$,

$k_{zi}=\sqrt{n_{i}^{2}k_{0}^{2}-k_{y}^{2}}$, (3)

$t_{ij}^{p}=\frac{2n_{i}n_{j}k_{zi}}{n_{j}^{2}k_{zi}+n_{i}^{2}k_{zj}}$, $t_{ij}^{s}=\frac{2k_{zi}}{k_{zi}+k_{zj}}$, (4)

$r_{ij}^{p}=\frac{n_{j}^{2}k_{zi}-n_{i}^{2}k_{zj}}{n_{j}^{2}k_{zi}+n_{i}^{2}k_{zj}}$, $r_{ij}^{s}=\frac{k_{zi}-k_{zj}}{k_{zi}+k_{zj}}$. (5)

The LDOS equations for infinitesimally thin films are shown below:

$\tilde{\rho}_{x}^{p}\left( \omega,k_{\|} \right)=\tilde{\rho}_{y}^{s}\left( \omega,k_{y} \right)=\tilde{\rho}_{z}^{s}\left( \omega,k_{y} \right)=0$, (6)

$\tilde{\rho}_{IP}^{s}\left( \omega,k_{\|} \right)=\left( \frac{1}{8\pi k_{0}^{2}} \right)\left( \frac{k_{0}}{k_{z3}} \right){|t_{31}^{s}|}^{2}$, (7)

$\tilde{\rho}_{IP}^{p}\left( \omega,k_{\|} \right)=\left( \frac{1}{8\pi k_{0}^{2}} \right)\left( \frac{k_{0}}{k_{z3}} \right){|\frac{k_{z1}}{n_{1}k_{0}}t_{31}^{p}|}^{2}$, (8)

$\tilde{\rho}_{OP}^{p}\left( \omega,k_{\|} \right)=\left( \frac{1}{8\pi k_{0}^{2}} \right)\left( \frac{k_{0}}{k_{z3}} \right){|\frac{k_{y}}{n_{1}k_{0}}\frac{n_{1}^{2}}{n_{2e}^{2}}t_{31}^{p}|}^{2}.$ (9)

In the model, we set the frequency ($\omega$) as a constant according to the exciton peak position. We assume that $\mu_{IP}(\omega)\text{=}\mu(\omega)\cdot\sin\alpha$, $\mu_{OP}(\omega)\text{=}\mu(\omega)\cdot\cos\alpha$, and the exciton PL comes from one dipole. $\mu(\omega)$ is the dipole moment of the emitter. *α* is the dipole orientation, which is defined as the angle between the dipole axis and the z-axis. $\mu_{IP}$ ($\mu_{OP}$) are the IP (OP) dipole moment components of the emitter obtained by dipole moment projection. The PL emission intensity $N^{s,p}(\omega,k_{||})$ is normalized in the end. For monolayer transition metal dichalcogenides (TMDs) and HBs, the dipole is located in an infinitesimally thin film, and the IP and OP refractive indices are taken as 2.12 and 1.70, respectively [14]. We assume that the dipole emits in the y-z plane so that *k*_||_ = *k*_y_.


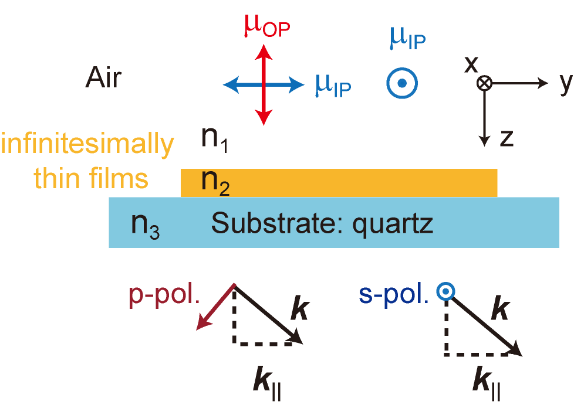


**Figure S6.** **Schematic of the geometry and the coordinate system.** The refractive indices n_1_, n_2_ and n_3_ are 1, 1.70, and 1.5, respectively.


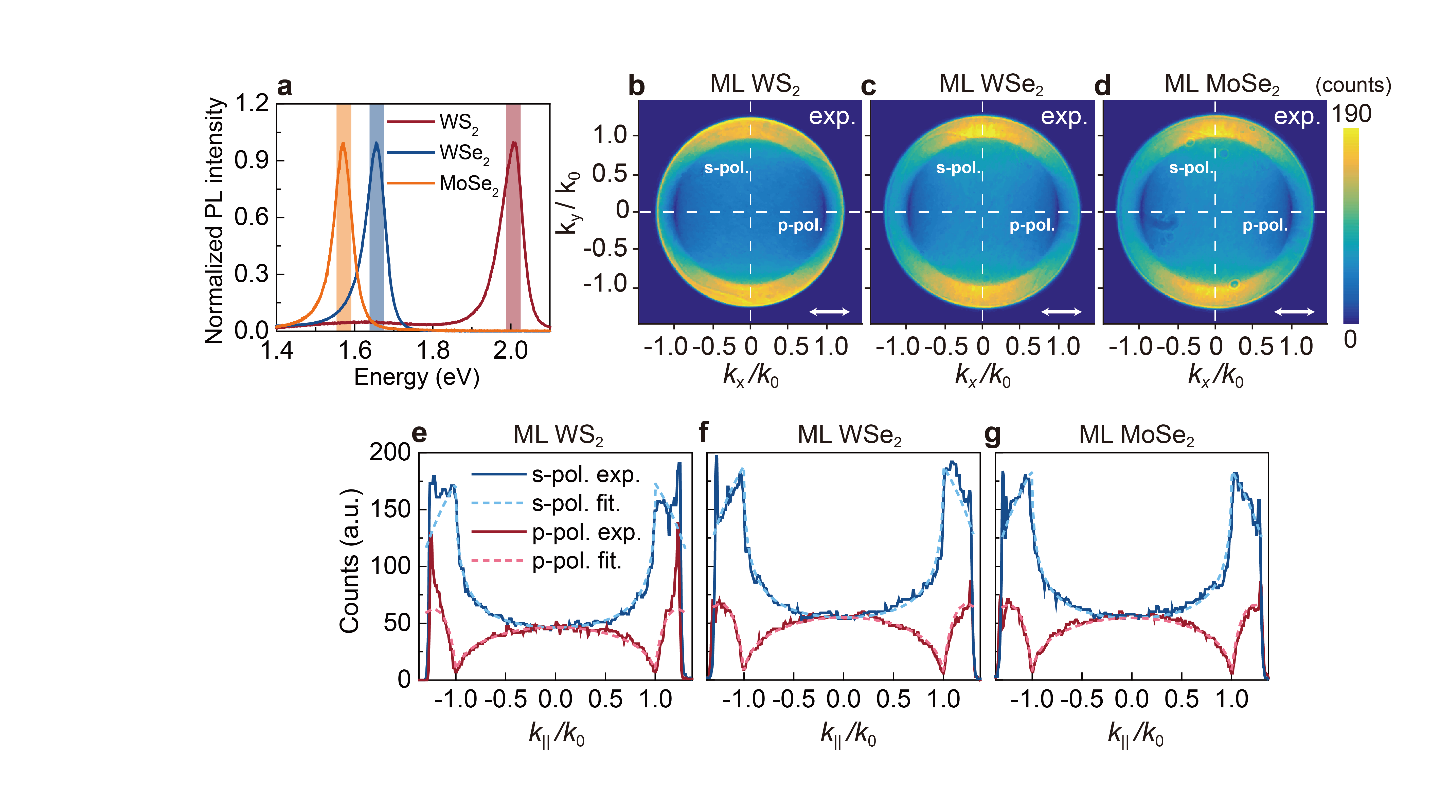


**Figure S7.** Normalized PL spectra (a) and Fourier images (b-d) of monolayer (ML) WS_2_, WSe_2_, and MoSe_2_. Fourier images were taken using a bandpass filter centered at the energy corresponding to the bright (A) exciton, with a bandwidth of ~13 nm. (e-g) Experimental and fitted cross-section lines of the bright excitons of the monolayers obtained from their Fourier images (b-d). The fitting results show the pure IP exciton character of all the excitons.

Figure S8a-d shows the normalized Fourier images of the dipole with different orientations. The dipole orientation (α) is defined as the angle between the dipole axis and the z-axis (Figure S8e). As seen from the cross-sections for p-polarization, when the dipole orientation is changed from 90° (IP) to 0° (OP), the emission intensity at *k*_||_ = *k*_0_ increases from the minimum to the maximum (Figure S8f). From the normalized Fourier image, we then extract the ratio R that equals the emission intensity ratio between p-polarization and s-polarization at *k*_||_ = *k*_0_. As seen, with an increase in the dipole orientation angle, the ratio R gradually decreases and reaches zero at 90° (Figure S8g). We find that the ratio R depends strongly on the OP refractive index but not on the wavelength. For example, the ratio R of the 45° dipole decreases with increasing OP refractive index (Figure S8h). In this work, the OP refractive index [14] of the HBs is taken as 1.70. Due to the small oscillator strength and technical difficulties, the refractive index of the TDE can hardly be obtained by experiments, especially the OP component.


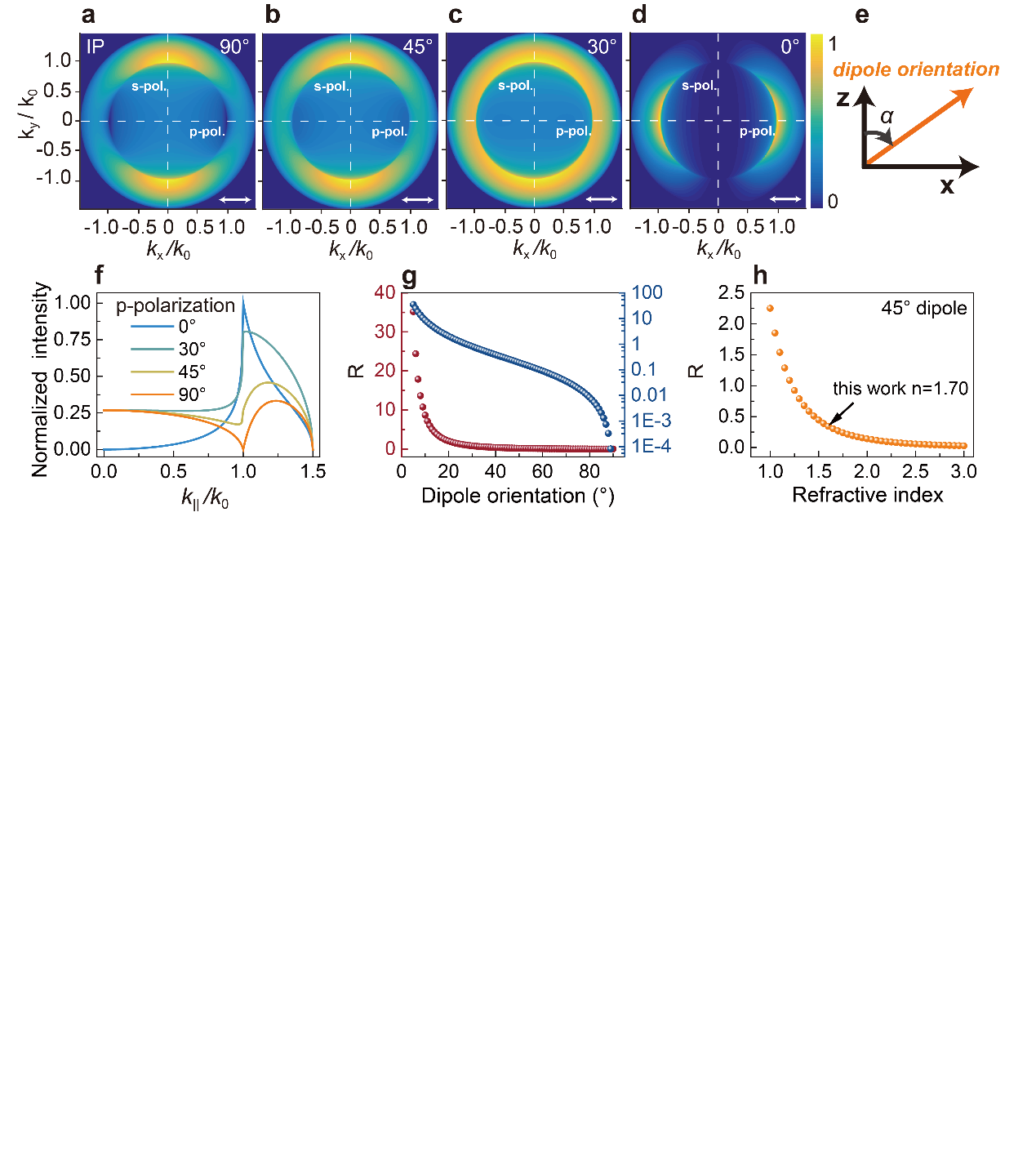


**Figure S8.** (a - d) Calculated x-polarized k-space emission patterns of 90° (IP), 45°, 30°, and 0° (OP) dipoles. The dipoles are located in an infinitesimally thin film, emitting at 1.35 eV with a refractive index of 1.70 and sitting on a quartz substrate (n=1.5). The white arrows and dashed lines denote the x-polarization direction and s-/p-polarization direction, respectively. (e) The dipole orientation α is defined as the angle between the dipole direction and the z-axis (vertical to the HB material plane). (f) Normalized emission intensity of 0°-90° dipoles for p-polarization. Ratio R as a function of dipole orientation (g) and refractive index (h).

In the simulation process for multilayer WS_2_ films, the IP and OP refractive indices are 2.12 and 1.70, respectively [14]. When imaging the *k*-space pattern of the highest energy PL peak in multilayer WS_2_, we find that R increases from 0.03 to almost 0.2 as the number of layers increases from 7 to 16 (Figure 4g). The fitting results show that the excitons of the 7- and 8-layer WS_2_ films are still IP (~90°) excitons, but the dipole orientations of the 11-, 14- and 16-layer WS_2_ films at approximately 1.95 eV are 83°, 84°, and 84°, respectively. These phenomena suggest that the OP exciton contribution increases when the thickness of WS_2_ increases. The IP exciton contribution still accounts for the majority of the intensity.


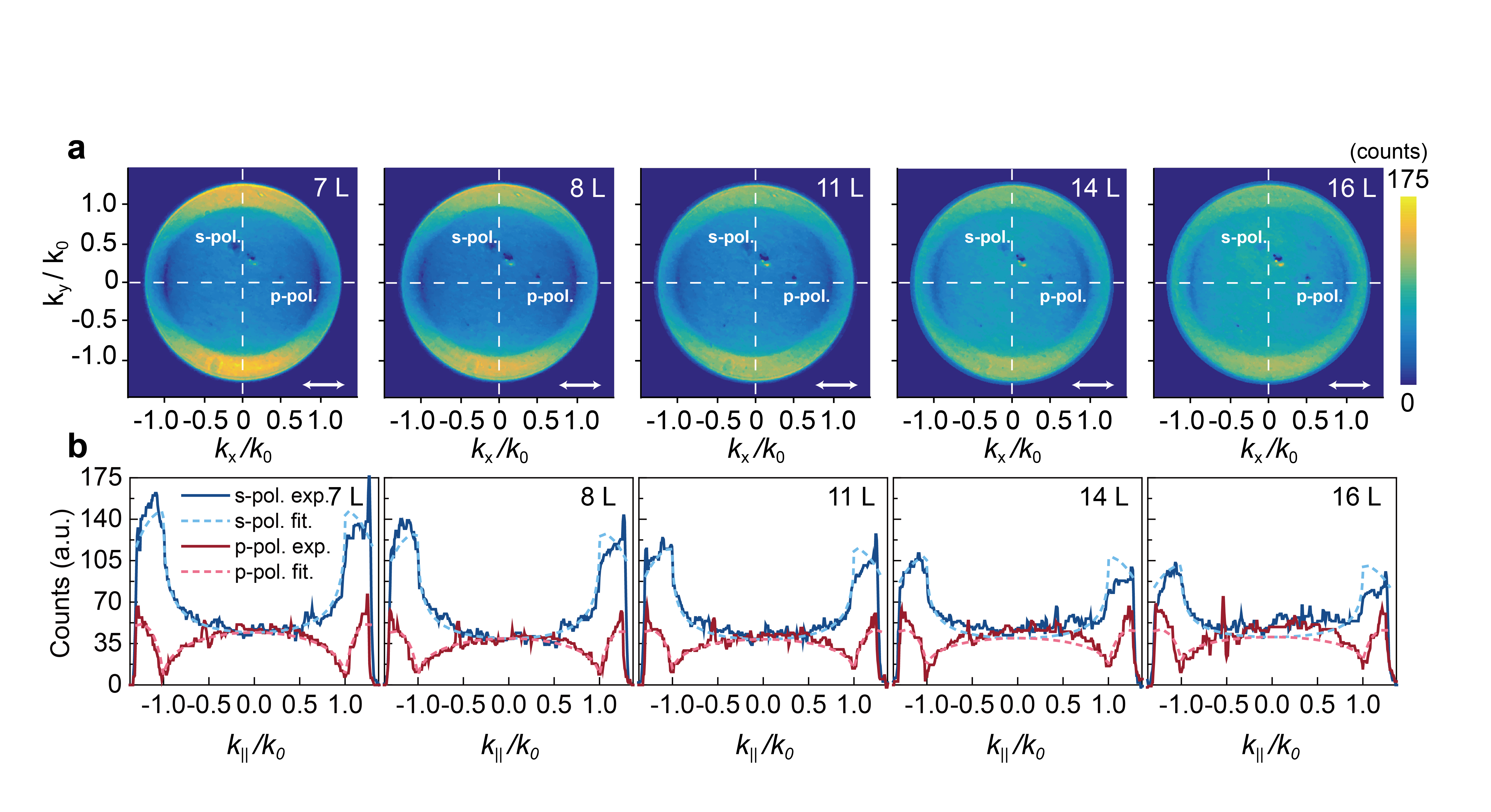


**Figure S9.** (a) *k*-space pattern of the exciton peak at approximately 1.95 eV shown in Figure 4f. A tunable bandpass filter with a bandwidth of 13 nm was used, and its bandpass position was centered at the corresponding exciton energy. (b) Corresponding experimental (solid lines) and fitted (dotted lines) data of the cross-sections for s- and p-polarization. The layer number of WS_2_ films is shown at the top right corner of the image.

## **II Supporting theoretical details**

### 2.1 Density functional theory (DFT) and GW calculation methods

The DFT calculations are performed with the Perdew, Burke, and Ernzerhof (PBE) functional, implemented in the QUANTUM ESPRESSO package [15,16]. The structure is relaxed with the van der Waals (vdW) DFT-D functional [17]. The ground state wavefunctions and eigenvalues are obtained from the DFT/PBE calculations with norm-conserving pseudopotentials. The plane-wave basis is set with a cutoff energy of 80 Ry with a 16 × 16 × 1 *k*-point grid. The vacuum space between neighboring layers is set to be more than 25 Å to avoid interactions between layers. Based on these parameters, the relaxed lattice constant and layer distance are 3.264 Å and 6.576 Å for AB-stacked WS_2_/WSe_2_. The excited-state properties of the heterostructure are calculated by the GW approximation within the general plasmon pole model, which is reliable in obtaining the excitonic properties of monolayer TMDs [18,19]. The unoccupied conduction band number involved in calculating the dielectric function and self-energy is approximately ten times the occupied valence band number. In solving the Bethe-Salpeter equation (BSE), we use a finer *k*-point grid of 32 × 32 ×1 for converged exciton states. All the GW-BSE calculations are performed with the BERKELEY GW code including the slab Coulomb truncation scheme to mimic suspended monolayer structures [20,21]. For optical absorption spectra, only the incident light polarized parallel to the plane is considered due to the depolarization effect.

### 2.2 Twisted WS_2_/WSe_2_ HBs

When two lattices are stacked on each other, a large periodic system, the moiré pattern, will emerge. For the case of twisted WS_2_/WSe_2_, the unit cells at different angles are shown in Figure S11. The supercell lattice vectors for the top WSe_2_ and bottom WS_2_ are

$T=n_{t}a_{1}+m_{t}a_{2}$, (10)

$B=n_{b}b_{1}+m_{b}b_{2}$, (11)

where $a_{1}$ and $a_{2}$ are the lattice vectors for the top layer, $b_{1}$ and $b_{2}$ are the lattice vectors for the bottom layer, and $n_{t}$, $m_{t}$, $n_{b}$, and $m_{b}$ are integers defined in Figure S10. These integers determine the size of the moiré pattern. Therefore, the commensurability condition is $\left|T\right|=\left|B\right|$ enforced by a small strain. The commensurate angle for twisted bilayer transition metal dichalcogenides is defined as

$\cos\theta=\frac{T*B}{\left|T\right|*\left|B\right|}$. (12)

The relaxed lattice constant for one monolayer of WS_2_ (WSe_2_) is 3.181 (3.315) Å, in line with previously reported values [22,23]. We build large twisted WS_2_/WSe_2_ HBs based on these relaxed monolayer systems. For twisted homobilayers, there is no strain. However, there is a small strain for twisted HBs. In our investigation, the strain for twisted HBs is always less than 0.50% (Table S2), which cannot significantly alter the electronic structures of the monolayers of WS_2_ (WSe_2_). All relevant structure parameters are presented in Table S2. Due to the hexagonal lattice of the TMD, each set of parameters describes a pair of twisted HBs, and the sum of the twist angles is equal to 60°.


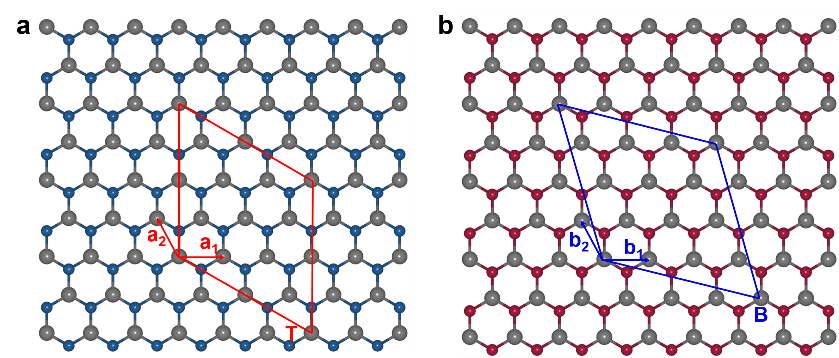


**Figure S10.** Schematics of a WS_2_/WSe_2_ supercell. The lattice vector of the top WSe_2_ supercell is $T=n_{t}a_{1}+m_{t}a_{2}$, where $a_{1}$ and $a_{2}$ are the primitive lattice vectors of WSe_2_ in (a). The lattice vector of the bottom WS_2_ supercell is $B=n_{b}b_{1}+m_{b}b_{2}$, where $b_{1}$ and $b_{2}$ are the primitive lattice vectors of WS_2_ in (b). $\theta$ is the twist angle between the rotated vectors *T* and *B*. The blue, red, and gray balls represent the Se, S, and W atoms, respectively.

**Table S2.** Parameters defining the commensurate supercells of WS_2_/WSe_2_ for each twist angle. The primitive-to-supercell scaling factors *n_t_*, *m_t_*, *n_b_* and *m_b_*, the strain in each layer, the number of atoms per HB supercell, and the length of the supercell basis vector (L) are listed. These factors are defined in Figure S10. For 0° and 60° samples, we list three sets of commensurate parameters under different strains. Limited by computational resources, we only consider the representative AA- and AB-stacking modes including 6 atoms.

|  | WSe_2_ | | WS_2_ | |  |  |
| --- | --- | --- | --- | --- | --- | --- |
| Twist angle (°) | (*n_t_*, *m_t_*) | Strain (%) | (*n_b_*, *m_b_*) | Strain (%) | Atoms | L (Å) |
| 0.0, 60.0 | (1,1) | 4.13 | (1,1) | 4.13 | 6 | 3.248 |
| 0.0, 60.0 | (14,14) | 2.76 | (15,15) | 2.76 | 1263 | 47.066 |
| 0.0, 60.0 | (20,20) | 0.70 | (21,21) | 0.70 | 2523 | 66.556 |
| 10.2, 49.8 | (2,4) | 0.48 | (1,5) | -0.48 | 177 | 17.628 |
| 16.1, 43.9 | (-4,1) | 0.07 | (1,3) | -0.07 | 75 | 11.477 |
| 28.1, 31.9 | (4,2) | 0.48 | (1,5) | -0.48 | 177 | 17.628 |


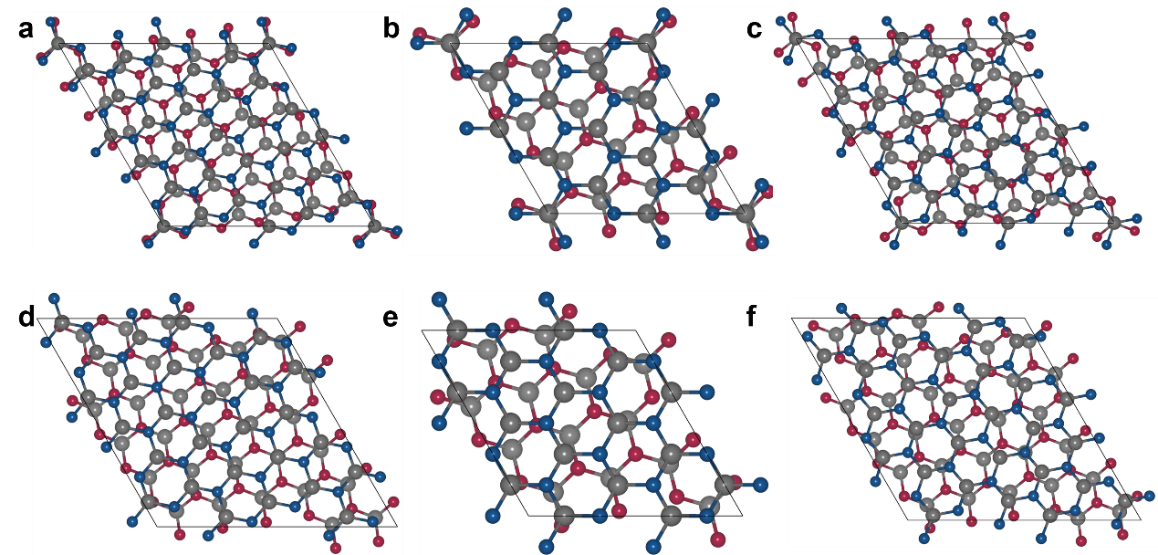


**Figure S11.** Atomic models of the WS_2_/WSe_2_ HBs with (a-c) an interlayer (parallel) twisted by a rotation angle of 10.2° (49.8°), 16.1° (43.9°), and 28.1° (31.9°), starting with AA stacking of WS_2_/WSe_2_ and with the rotation center at the W atom. (d-f) Atomic structures of the WS_2_/WSe_2_ HBs with an interlayer (parallel) twisted by a rotation angle of 10.2° (49.8°), 16.1° (43.9°), and 28.1° (31.9°), starting with AB stacking of WS_2_/WSe_2_. WSe_2_ is the top layer, and WS_2_ is the bottom layer. The blue, red, and gray balls represent the Se, S, and W atoms, respectively.

There are many ways to obtain twisted bilayer TMDs, for example, by using different initial structures and twist centers. Different structures with the same twist angles lead to fluctuations for a single angle in experiments. Here, we choose the AA- and AB-stacking modes, with the highest symmetry, as our initial configurations. For the AA-stacking mode, we can easily choose the metal atom as the twist center. For the AB-stacking mode, we set the twist center at the hexagonal center.


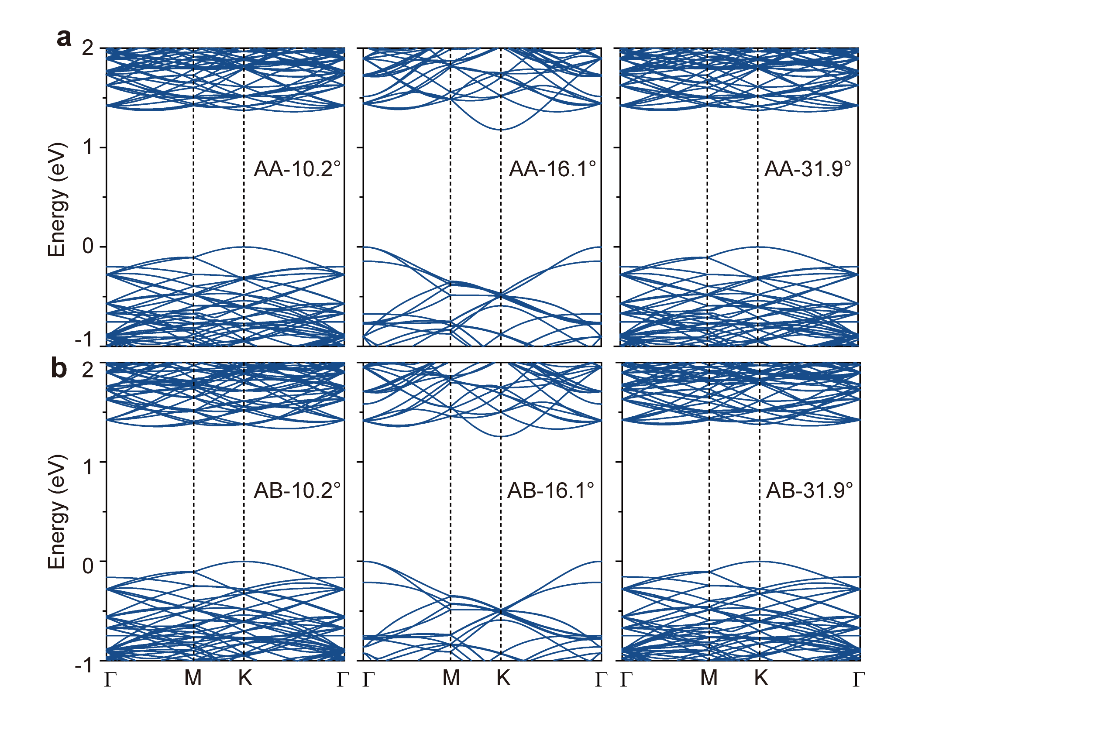


**Figure S12.** Band structures for each twisted bilayer of WS_2_/WSe_2_ in Figure S9. For the same twist angle, there is almost no difference in the band edge between the different twist structures, similar to the case of AA and AB primitive WS_2_/WSe_2_. The direct bandgaps in (a) are 1.379 eV (K-K), 1.413 eV (Γ-Γ), and 1.379 eV (K-K), from left to right. Because the direct bandgaps correlate with the optical transitions, we present the direct bandgaps for different twist angles in the main text. There is a sharp increase in the bandgap (~0.6 eV) from the primitive cell to the twisted sample. For all the twisted supercells, the bandgap does not change much with the angle. This phenomenon also appears in other twisted bilayer structures [24,25].

Considering the large strain (4.13%) of small unit cell WS_2_/WSe_2_ with 6 atoms, we enlarge the supercell (2523 atoms) with small strain (0.7%) and calculate the energy gap by SIESTA [26], which is much faster than the QUANTUM ESPRESSO package because of the different bases. With increasing strain, the bandgap increases from 0.92 eV to 1.37 eV. This phenomenon also appears in other TMD heterostructures [27]. In contrast, when using the unstrained band alignment to obtain the bandgap of WS_2_/WSe_2_ without strain [28], the bandgap at 0° and 60° is 1.11 eV [29].


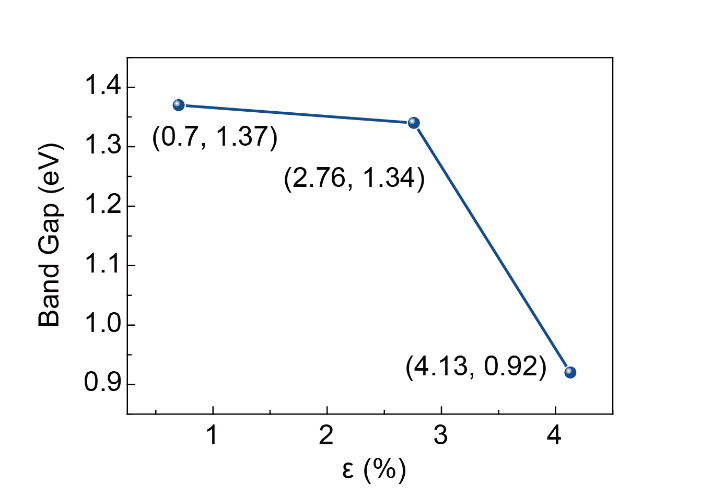
**Figure S13.** Computed electronic bandgaps (PBE) of WS_2_/WSe_2_ bilayers at a twist angle of 0° (60°) for different strains. For the same angle, the larger the strain is, the smaller the system is. This shows that the bandgap decreases with increasing strain.

To analyze the origin of the optical transition modulated by the vdW interlayer interaction, we calculated the projected band structure of the HB. The Brillouin zones of an individual monolayer are twisted with respect to the other monolayer in twisted HB bilayers, and their bands are folded into the Brillouin zones of the twisted supercell. This indicates that the K-points of the monolayer may be folded onto other k-points in the twisted supercell. In detail, Figure S14 shows two cases (the color arrows indicate the relevant optical transitions in the HB), i.e., 10.2° HB: K_WSe2_→K_HB_, K_WS2_→K_HB_, Γ_hybrid_→Γ_HB_; 16.1° HB: K_WSe2_→Γ_HB_, K_WS2_→K_HB_, Γ_hybrid_→Γ_HB_. Here, we focus on the optical transitions, which are affected by the vdW interlayer interaction. The two relevant transitions K-K and Γ-K of the HBs with different angles are shown in Figure 2f. Both K-K and Γ-K transitions present twist-angle-dependent properties. Compared to the K-K transition, the trend of the Γ-K transition is more consistent with the experimental TDE results.


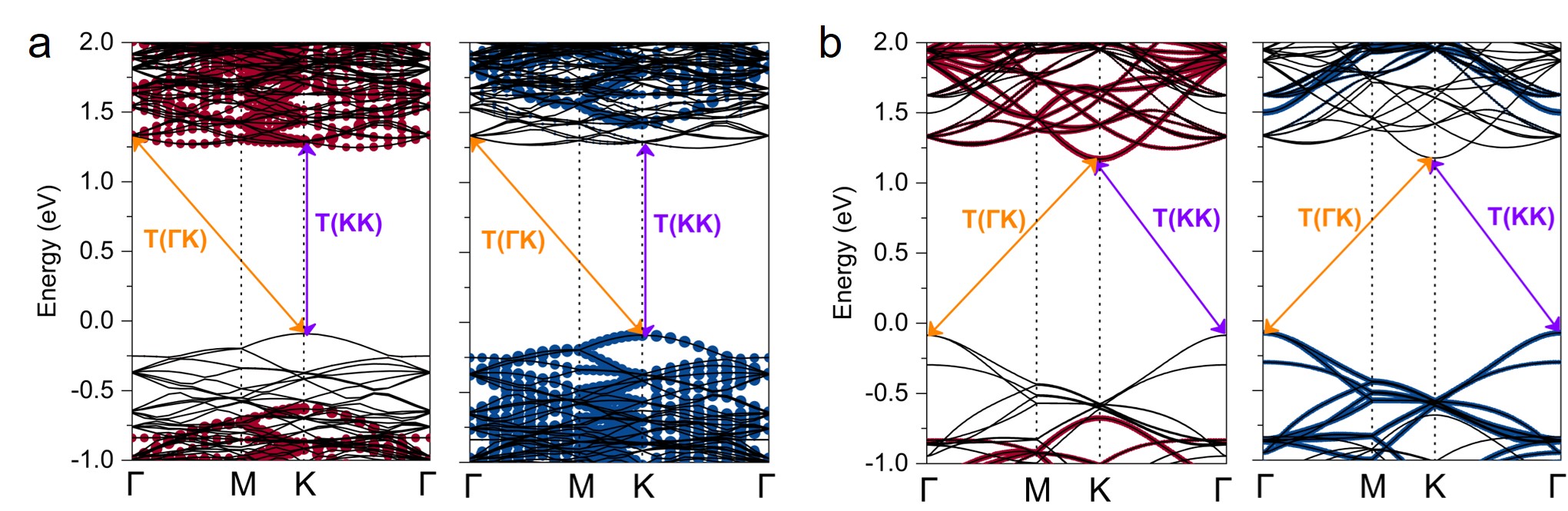


**Figure S14.** DFT band structures of HBs with twist angles of 10.2° (a) and 16.1° (b). The colors indicate the contributions from the WS_2_ layer (red) and WSe_2_ (blue) layer.

### 2.3 Electronic structures of AA- and AB-stacked HBs


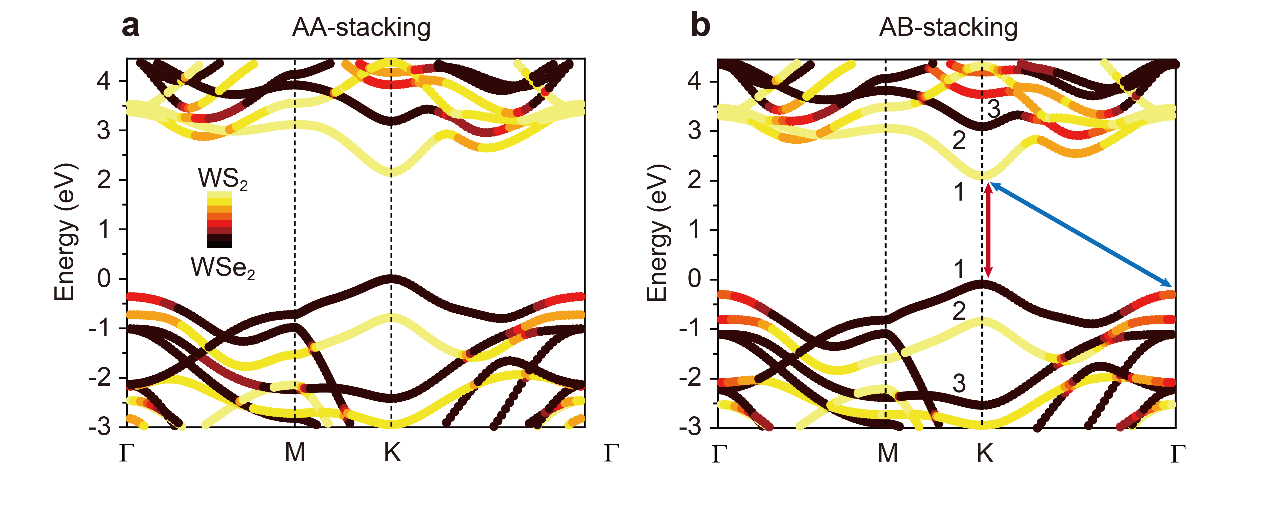


**Figure S15.** (a-b) Band structures of AA- and AB-stacked WS_2_/WSe_2_ bilayers. The band index is labeled. There is no difference between the band edges of the two band structures, and the bandgap is 0.780 (2.174) eV at the PBE (GW) level. This phenomenon also appears in other studies [30].

### 2.4 Optical transitions

#### 2.4.1 Moiré pattern


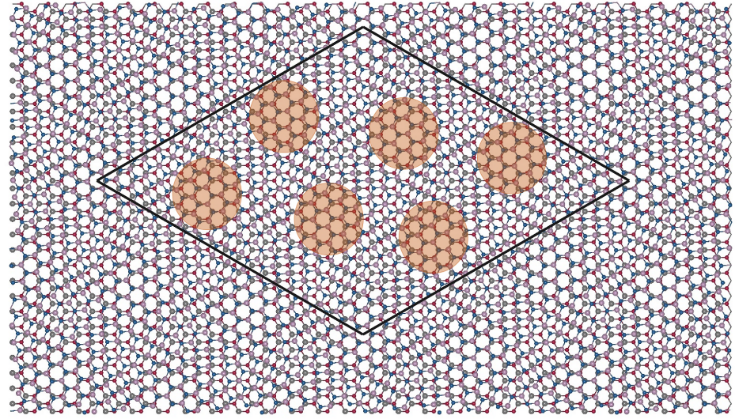
There are six high-symmetry configurations [31] for primitive bilayer TMDs. The formed moiré patterns usually contain many local atomic configurations [4,32]. All the local atomic structures contribute to the experimental optical transitions. Due to the computational complexity of Berkeley GW, we select the AA- and AB-stacking configurations and calculate their optical absorbance. Then, we average the optical absorbance of the AA and AB configurations and compare the average optical absorbance with the experimental PL spectrum. However, the Berkeley GW code does not include the indirect optical transition because it does not consider phonon effects. To check the indirect optical transition, we calculate the supercell of $\sqrt{3}\times\sqrt{3}$ WS_2_/WSe_2_, in which the K point has been folded onto the Γ point, to include the Γ-K indirect transition. If there is no indirect transition, then there are no new absorbance peaks in the supercell compared with the absorbance of the primitive bilayer.

**Figure S16.** Different local atomic alignments in a WS_2_/WSe_2_ vertical heterostructure with a small twist angle (moiré pattern). Highlighted regions correspond to local atomic configurations with high symmetry.

####
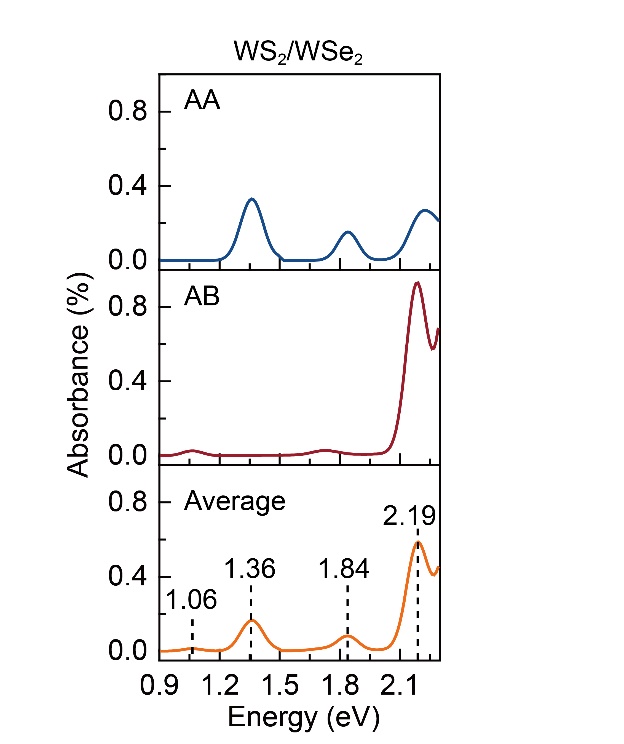
2.4.2 Optical absorbance

**Figure S17.** To include the indirect optical transition (Γ-K), we present the optical absorption of $\sqrt{3}\times\sqrt{3}$ AA- and AB-stacked WS_2_/WSe_2_ and their average result at the bottom. Notably, the intralayer exciton binding energy of the heterostructure should be different from that of an isolated monolayer sample as a result of interlayer hopping and changes in dielectric screening arising from the other layer.

#### 2.4.3 Joint density of excited states of WS_2_/WSe_2_

The joint density of states function $J_{if}\left( \xi\right)$ is defined as

$J_{if}\left( \xi\right)=\sum_{\vec{k},\vec{s}} \delta(\xi-\varepsilon_{f}\left( \vec{k} \right)-|\varepsilon_{i}\left( \vec{k} \right)|)$;$\xi=\hbar\omega$, (13)

where the dispersion relations are given in the form

$\varepsilon_{f}=\varepsilon_{f}\left( \vec{k} \right)$, (14)

$\varepsilon_{i}={-\varepsilon}_{i}\left( \vec{k} \right)$, (15)

where *k* is the wavevector, and s is the spin. $\varepsilon_{i}$ and $\varepsilon_{f}$ are the eigenvalues for the initial valence band states $|i,k,s\rangle$ and the final conduction band states $|f,k,s\rangle$ involved in the transitions. The joint density of states function therefore only includes the direct transition because the definition focuses on the same *k* point. In Figure S18, we can see that there is a peak at approximately 1.2 eV, indicating a direct transition. There is no peak at 1.4 eV in the figure, meaning that there is no direct transition. If we observe a peak at 1.4 eV, then it must originate from an indirect transition.


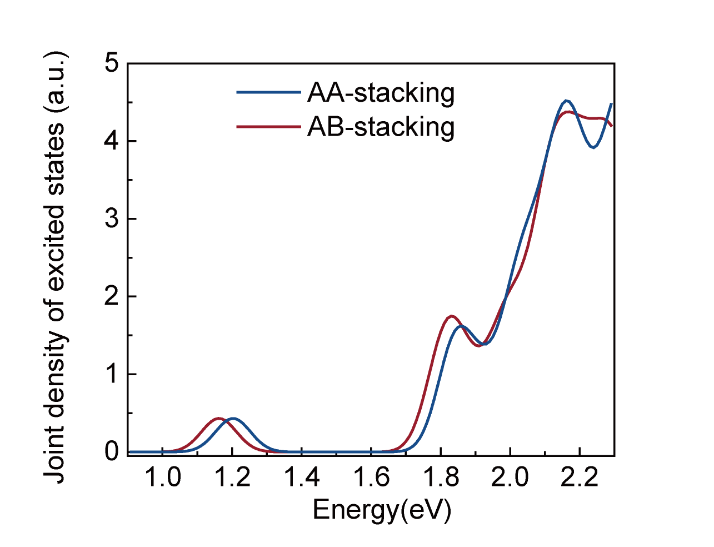


**Figure S18.** Calculated joint density of excited states for primitive AA- (blue line) and AB-stacked (red line) WS_2_/WSe_2_. Only direct optical transitions are included in this calculation [7,33].

#### 2.4.4 Excitonic weight analysis

**Table S3.** Transition analysis of the excitonic weight A_vck_ in a bilayer of WS_2_/WSe_2_. The valence and conduction band indices have been labeled in the projected band structure.

| Exciton state (eV) | Conduction band | Valence band | K-points | A_vck_ |
| --- | --- | --- | --- | --- |
| 1.06 | 1 | 1 | K | 0.99929 |
| 1.36 | 1 | 1 | K/Γ | 0.99700 |
| 1.84 | 1 | 2 | K | 0.96220 |
| 2.19 | 2 | 1 | K | 0.90100 |

The two-particle electron-hole wavefunction is obtained by solving the BSE:

$H^{(eff)}|A_{\lambda}\rangle=E_{\lambda}|A_{\lambda}\rangle$. (16)

The eigenvectors result from the diagonalization of the BSE, i.e., $|A_{\lambda}\rangle=\Sigma_{\upsilon ck}A_{\upsilon ck}^{\lambda}|\upsilon ck\rangle$, where the coefficient $A_{\upsilon ck}^{\lambda}=\langle\upsilon ck|A^{\lambda}\rangle$is the excitonic weight. It enters into the expression of the two-particle electron-hole wavefunction:

$\Psi_{\lambda}(\mathrm{r}_{h},\mathrm{r}_{e})=\mathop{\Sigma}\limits_{\upsilon\text{ck}} A_{\upsilon ck}^{\lambda}\psi_{\upsilon k}(\mathrm{r}_{h})\psi_{\upsilon k}^{*}(\mathrm{r}_{e})$. (17)

The excitonic weight defines the transitions between the quasi-particle wavefunctions $\psi_{\upsilon k}(\mathrm{r}_{h})$ and $\psi_{\upsilon k}^{*}(\mathrm{r}_{e})$. $\Psi_{\lambda}(\mathrm{r}_{h},\mathrm{r}_{e})$ depends on the electron and hole coordinates. The two-particle electron-hole wavefunction can be visualized in real space if we fix the hole coordinates.

**References**

1. Seyler K L, Rivera P, Yu H Y *et al.* Signatures of moiré-trapped valley excitons in MoSe_2_/WSe_2_ heterobilayers. *Nature* 2019; **567**: 66-70.

2. Rivera P, Seyler K L, Yu H Y *et al.* Valley-polarized exciton dynamics in a 2D semiconductor heterostructure. *Science* 2016; **351**: 688-91.

3. Yu H, Liu G-B, Tang J *et al.* Moiré excitons: from programmable quantum emitter arrays to spin-orbit–coupled artificial lattices. *Sci Adv* 2017; **3**: e1701696.

4. Tran K, Moody G, Wu F *et al.* Evidence for moiré excitons in van der Waals heterostructures. *Nature* 2019; **567**: 71-5.

5. Nayak P K, Horbatenko Y, Ahn S *et al.* Probing evolution of twist-angle-dependent interlayer excitons in MoSe_2_/WSe_2_ van der Waals heterostructures. *ACS Nano* 2017; **11**: 4041-50.

6. Miller B, Steinhoff A, Pano B *et al.* Long-lived direct and indirect interlayer excitons in van der Waals heterostructures. *Nano Lett* 2017; **17**: 5229-9.

7. Hanbicki A T, Chuang H J, Rosenberger M R *et al.* Double indirect interlayer exciton in a MoSe_2_/WSe_2_ van der Waals heterostructure. *ACS Nano* 2018; **12**: 4719-26.

8. Kunstmann J, Mooshammer F, Nagler P *et al.* Momentum-space indirect interlayer excitons in transition-metal dichalcogenide van der Waals heterostructures. *Nat Phys* 2018; **14**: 801-5.

9. Karni O, Barré E, Lau S C *et al.* Infrared interlayer exciton emission in MoS_2_/WSe_2_ heterostructures. *Phys Rev Lett* 2019; **123**: 247402.

10. Alexeev E M, Ruiz-Tijerina D A, Danovich M *et al.* Resonantly hybridized excitons in moiré superlattices in van der Waals heterostructures. *Nature* 2019; **567**: 81-6.

11. Jin C, Regan E C, Wang D *et al.* Identification of spin, valley and moiré quasi-angular momentum of interlayer excitons. *Nat Phys* 2019; **15**: 1140-4.

12. Hsu W T, Zhao Z A, Li L J *et al.* Second harmonic generation from artificially stacked transition metal dichalcogenide twisted bilayers. *ACS Nano* 2014; **8**: 2951-8.

13. Schuller J A, Karaveli S, Schiros T *et al.* Orientation of luminescent excitons in layered nanomaterials. *Nat Nanotechnol* 2013; **8**: 271-6.

14. Thilagam A. Exciton complexes in low dimensional transition metal dichalcogenides. *J Appl Phys* 2014; **116**: 053523.

15. Giannozzi P, Baroni S, Bonini N *et al.* QUANTUM ESPRESSO: a modular and open-source software project for quantum simulations of materials. *J Phys: Condens Matter* 2009; **21**: 395502.

16. Perdew J P, Burke K and Ernzerhof M. Generalized gradient approximation made simple *Phys Rev Lett* 1997; **78**: 1396.

17. Barone V, Casarin M, Forrer D *et al.* Role and effective treatment of dispersive forces in materials: Polyethylene and graphite crystals as test cases. *J Comput Chem* 2009; **30**: 934-9.

18. Qiu D Y, da Jornada F H and Louie S G. Optical spectrum of MoS_2_: many-body effects and diversity of exciton states. *Phys Rev Lett* 2013; **111**: 216805.

19. Aidelsburger M, Atala M, Lohse M *et al.* Realization of the hofstadter hamiltonian with ultracold atoms in optical lattices. *Phys Rev Lett* 2013; **111**: 185301.

20. Deslippe J, Samsonidze G, Strubbe D A *et al.* BerkeleyGW: A massively parallel computer package for the calculation of the quasiparticle and optical properties of materials and nanostructures. *Comput Phys Commun* 2012; **183**: 1269-89.

21. Ismail-Beigi S. Truncation of periodic image interactions for confined systems. *Phys Rev B* 2006; **73**: 233103.

22. Shi H L, Pan H, Zhang Y W *et al.* Quasiparticle band structures and optical properties of strained monolayer MoS_2_ and WS_2_. *Phys Rev B* 2013; **87**: 155304.

23. Chang C H, Fan X F, Lin S H *et al.* Orbital analysis of electronic structure and phonon dispersion in MoS_2_, MoSe_2_, WS_2_, and WSe_2_ monolayers under strain. *Phys Rev B* 2013; **88**: 195420.

24. Wang Z L, Chen Q and Wang J L. Electronic structure of twisted bilayers of graphene/MoS_2_ and MoS_2_/MoS_2_. *J Phys Chem C* 2015; **119**: 4752-58.

25. Lu N, Guo H, Zhuo Z *et al.* Twisted MX_2_/MoS_2_ heterobilayers: effect of van der Waals interaction on the electronic structure. *Nanoscale* 2017; **9**: 19131-8.

26. Soler J M, Artacho E, Gale J D *et al.* The SIESTA method for ab initio order-N materials simulation. *J Phys: Condens Matter* 2002; **14**: 2745-79.

27. Lu N, Guo H, Li L *et al.* MoS_2_/MX_2_ heterobilayers: bandgap engineering via tensile strain or external electrical field. *Nanoscale* 2014; **6**: 2879-86.

28. Lu X, Li X and Yang L. Modulated interlayer exciton properties in a two-dimensional moiré crystal. *Phys Rev B* 2019; **100**: 155416.

29. Liang Y, Huang S, Soklaski R *et al.* Quasiparticle band-edge energy and band offsets of monolayer of molybdenum and tungsten chalcogenides. *Appl Phys Lett* 2013; **103**: 042106.

30. Liu Q H, Li L Z, Li Y F *et al.* Tuning electronic structure of bilayer MoS_2_ by vertical electric field: a first-principles investigation. *J Phys Chem C* 2012; **116**: 21556-62.

31. He J, Hummer K and Franchini C. Stacking effects on the electronic and optical properties of bilayer transition metal dichalcogenides MoS_2_, MoSe_2_, WS_2_ and WSe_2_. *Phys Rev B* 2014; **89**: 075409.

32. Naik M H and Jain M. Ultraflatbands and shear solitons in moiré patterns of twisted bilayer transition metal dichalcogenides. *Phys Rev Lett* 2018; **121**: 266401.

33. Cabrera C I, Contreras-Solorio D A and Hernandez L. Joint density of states in low dimensional semiconductors. *Physica E* 2016; **76**: 103-8.
